# Large-scale coherent Ising machine based on optoelectronic parametric oscillator

**DOI:** 10.1038/s41377-022-01013-1

**Published:** 2022-11-25

**Authors:** Qizhuang Cen, Hao Ding, Tengfei Hao, Shanhong Guan, Zhiqiang Qin, Jiaming Lyu, Wei Li, Ninghua Zhu, Kun Xu, Yitang Dai, Ming Li

**Affiliations:** 1 State Key Laboratory on Integrated Optoelectronics, Institute of Semiconductors, Chinese Academy of Sciences, Beijing, China; 2 School of Electronic, Electrical and Communication Engineering, University of Chinese Academy of Sciences, Beijing, China; 3 Center of Materials Science and Optoelectronics Engineering, University of Chinese Academy of Sciences, Beijing, China; 4 State Key Laboratory of Information Photonics and Optical Communications, Beijing University of Posts and Telecommunications, Beijing, China; 5 School of Optical-Electrical and Computer Engineering, University of Shanghai for Science and Technology, Shanghai, China; 6 Peng Cheng Laboratory, Shenzhen, China

**Keywords:** Microwave photonics, Optoelectronic devices and components

## Abstract

Ising machines based on analog systems have the potential to accelerate the solution of ubiquitous combinatorial optimization problems. Although some artificial spins to support large-scale Ising machines have been reported, e.g., superconducting qubits in quantum annealers and short optical pulses in coherent Ising machines, the spin stability is fragile due to the ultra-low equivalent temperature or optical phase sensitivity. In this paper, we propose to use short microwave pulses generated from an optoelectronic parametric oscillator as the spins to implement a large-scale Ising machine with high stability. The proposed machine supports 25,600 spins and can operate continuously and stably for hours. Moreover, the proposed Ising machine is highly compatible with high-speed electronic devices for programmability, paving a low-cost, accurate, and easy-to-implement way toward solving real-world optimization problems.

## Introduction

Combinatorial optimization problems can be found everywhere in modern society, such as drug discovery^1,2^, finance^3^, traffic flow optimization^4^, and machine learning^5^. Many combinatorial optimization problems are classified as the non-deterministic polynomial-time (NP)-hard or NP-complete complexity classes; these are difficult to be solved on standard digital computers because the number of combinations grows exponentially or factorially as the problem size *N* increases. Although some software algorithms, such as simulated annealing and other approximate algorithms^6–9^, have been developed to accelerate the computation, using a Von Neumann architecture to solve these problems remains time-consuming due to the step-by-step computation mode and limited operating frequency^10^. Interestingly, the solution to these problems can be effectively accelerated by mapping them onto an analog Ising machine. The Hamiltonian of an *N*-spin Ising machine without an external field is given by $H = - \mathop {\sum}\nolimits_{1 \le i < j \le N} {J_{i,j}\sigma _i\sigma _j}$, where *J*_*i,j*_ is the spin interaction between the *i-*th and *j-*th spins, and *σ*_*i*_ and *σ*_*j*_ respectively denote the *z* projection of the spins with eigenvalues of either 1 or −1. Finding the optimal answer is equivalent to searching the ground state of an Ising machine^11^, and computational acceleration is achieved by utilizing the intrinsic convergence property of the system, as convergence to the ground state often occurs at high speed.

Large-scale, stable, and programable Ising machines are highly desired to solve real-world optimization problems^1–5^. Many Ising machines, e.g., those based on trapped ions^12,13^, superconducting circuits^14–17^, molecules^18,19^, optical^20–33^, optoelectronic^34^, and electrical systems^35–38^, have been reported. The optimization principle of most types of these Ising machines is based on the idea of minimum power dissipation^39^. Among them, coherent Ising machines (CIMs) using degenerate optical parametric oscillators (DOPOs) have drawn a substantial amount of attention due to their attractive advantages, such as large scale^22,23^ and flexible connectivity^24,30,33^. It has also been reported that DOPO-based Ising machines are superior to quantum annealers^32^ and digital computers^24,33^ in some specific optimization problems. However, as the artificial spins are represented by the phases of the short optical pulses and stored in a long fiber cavity, the stability is weak because the optical phase is very sensitive to the cavity delay variation^24^. According to $\Delta \varphi = 2\pi f_o\Delta t$, where $\Delta \varphi$ is the phase change of the optical pulse, $f_o$ is the optical frequency (around 200 THz), and $\Delta t$ is the time jitter, a few femtoseconds of jitter would reverse the spin sign, causing the Hamiltonian to evolve away from the expectation.

Here, we propose a large-scale, stable Ising machine based on an optoelectronic parametric oscillator (OEPO)^40^. In the proposed CIM, the artificial Ising spins are represented by the phases of the short microwave pulses generated in an optoelectronic cavity. The number of generated spins is as large as 25,600, and the machine stably operates over 12 h. One-dimensional (1D) and two-dimensional (2D) Ising model simulations were conducted on the proposed CIM, and the experimental results suggest the machine has promising potential in finding the low-energy state. In addition, the machine was used to solve max-cut problems, which are well-known NP-hard problems, and a high success rate of finding the maximum cut was achieved.

## Results

### Principle and experimental setup

**Fig. 1:**
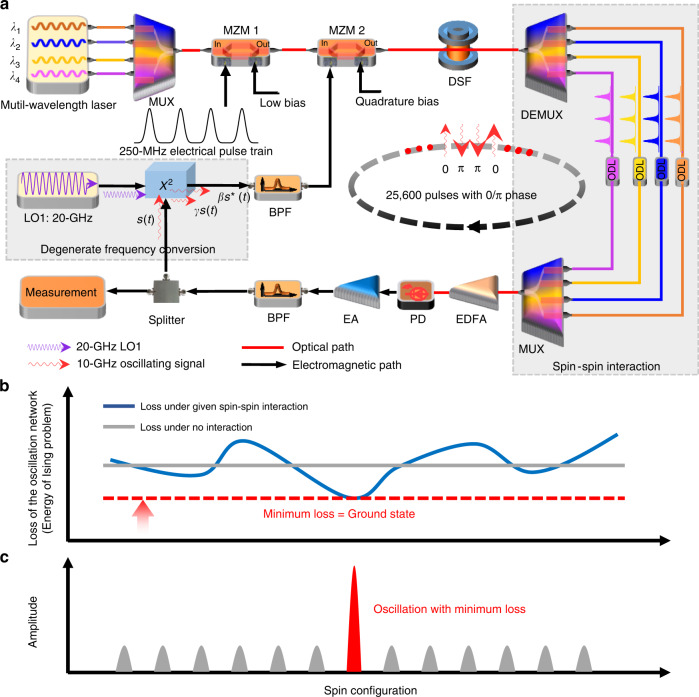
The schematic diagram and operation principle of the proposed OEPO-based Ising machine. **a** The schematic diagram. Spins are represented as binary phases of short microwave pulses generated from the OEPO, and spin interactions are realized by using different channels in the optical path with configurable delays. *MZM* Mach-Zehnder modulator, *DSF* dispersion-shifted fiber, *MUX* multiplexer, *ODL* optical delay line, *DEMUX* demultiplexer, *EDFA* Erbium-doped fiber amplifier, *PD* photodetector, *EA* electrical amplifier, *BPF* bandpass filter, *LO* local oscillator. **b** A given spin-spin interaction would change the global network loss, and the minimum-loss operation can be achieved by gradually increasing the gain of the OEPO network. **c** The network with the minimum-loss spin configuration has the maximum possibility to oscillate, and the phase configuration corresponds to the answer to a given Ising problem

Figure 1 presents the experimental setup and operation principle of the OEPO-based Ising machine. The OEPO was used to generate continuous wideband or single-frequency microwave signals with uncertain frequency and phase^40^. In the proposed machine, the oscillations in the OEPO cavity are discrete pulses with half the frequency of a local oscillation and locked with a relative 0 or *π* phase via an electric mixer (see section 1 in Supplementary Materials). The function of the electric mixer is similar to that of the nonlinear crystal in the DOPO. The binary-phase oscillation can then be used to simulate an artificial Ising spin, e.g., the 0-phase/*π*-phase state represents up/down spin.

With the help of time-division multiplexing, large-scale, discrete microwave pulse oscillations can be obtained in a single long fiber cavity. In such an oscillation network, the spin-spin interaction can be implemented by delay lines^21,22^ or a measurement-feedback circuit^24,30,33^. The delay-line scheme, implemented by a wavelength-division multiplexing (WDM) system and tunable optical delay lines (ODLs), is adopted in our demonstration. The oscillating microwave signal is distributed to different optical channels using the WDM as a beam splitter. Each channel propagates through a specific delay line (with an *n*-bit delay). These channels are combined using another WDM as a beam adder and launched into the photodetector (PD); thus, the *i*-th spin and the (*i* + *n*)-th spin interact. In this scheme, the coupling strength is controlled by the corresponding channel’s laser power, and the coupling sign can be reversed by tuning the optical delay line.

A specific spin-spin interaction, defined by matrix ***J***, would change the global loss of the OEPO network and result in the corresponding phase configuration (see section 1 in Supplementary Materials). Phase configuration with minimum loss has the maximum possibility to be selected and oscillate stably^20^. The OEPO network can operate around the minimum-loss state and outputs the corresponding microwave signal whose phase configuration corresponds to the optimal solution of the given Ising problem.

### Generation of large-scale, stable artificial spins

**Fig. 2:**
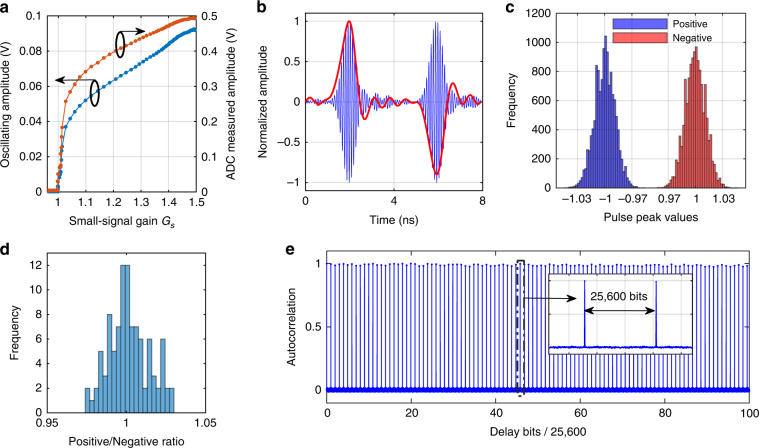
The experimental results of non-interaction oscillation. **a** The amplitudes of the OEPO output and the ADC measurement as functions of the cavity small-signal gain. **b** The oscillating microwave pulses centered at 10 GHz (blue curve), along with the corresponding demodulated baseband waveforms (red curve). **c** The histogram shows the frequency of normalized peaks of the demodulated baseband pulses. The obvious distinction between positive and negative baseband pulses suggests that microwave pulses oscillate with a relative phase of either 0 or *π*. This binary phase oscillation is used to represent the Ising spin. **d** The histogram shows the frequency of the ratio of positive pulses to negative pulses in 100 tests. Each test restarted from the noise state to the stable state. **e** The autocorrelation of the pulse peaks. Inset: zoomed-in view of the area of the red dashed rectangle. The peaks repeat each 25,600-bit delay, indicating that the optoelectronic cavity stores 25,600 microwave pulses. The flat peaks suggest that the OEPO operates stably

The large scale and high stability of the proposed system were verified in a non-interaction (***J*** is a unit matrix) artificial spin network. The results are exhibited in Fig. 2. The oscillation frequency of the microwave pulse centers at 10 GHz, and the wavelength is about 2 cm in the optical fiber with a refractive index of 1.47. The microwave pulse repetition period/rate is 4 ns/250 MHz; thus, 25,600 spins are obtained in a 20-km fiber. Because the ratio between the cavity length and the oscillation wavelength is dramatically decreased from 10^9^ to 10^6^, the artificial spins in the OEPO are much less sensitive to temperature fluctuation and ambient vibration, yielding much higher stability compared to the DOPO counterpart.

The oscillating amplitude as a function of the cavity small-signal gain is presented in Fig. 2a. When the cavity small-signal gain is below the OEPO threshold, the oscillator is in the noise state; when the gain is near the threshold, the curve exhibits a sharp shape. As the gain continuously increases, the amplitude increases slowly. Another external microwave source (LO2), which is synchronized to LO1 and has the same frequency as the oscillating signal, is used to demodulate the short microwave pulses to extract the microwave spin phase (see section 2 in Supplementary Materials).

Two oscillating microwave pulses with relative 0 and *π* phases, along with their demodulated baseband pulses, are shown in Fig. 2b. The histogram of the peak values presented in Fig. 2c indicates clear 0/*π*-phase oscillation. Because oscillation begins from shot noise and thermal noise, each microwave pulse is independent, and the global phase configuration would be random if no interaction is implemented. One hundred tests were performed, and the ratios between the positive and negative peaks ranged from 0.97 to 1.03, suggesting the equal probability of 0-phase and *π*-phase oscillations.

Millions of demodulated pulse peaks were recorded, and the autocorrelation was calculated. The periodic peaks were repeated at a delay of 25,600 pulses, suggesting that 25,600 spins were generated in the optoelectronic cavity. The stable delta sequence suggests that the microwave pulses maintained their amplitude and phase after each roundtrip.

### Simulations of the Ising model

To implement a 1D Ising simulation, a second channel with an additional 1-bit delay was used to interact with the neighboring spins. The formed 1D Ising model was a closed loop, and the coupling was unidirectional (*J*_*i-1,i*_ ≠ 0, *J*_*i,i-1*_ = 0) from the (*i*-1)-th to the *i*-th spin for *i* ≤ 25,600 and from the 25,600-th to the first spin.

**Fig. 3:**
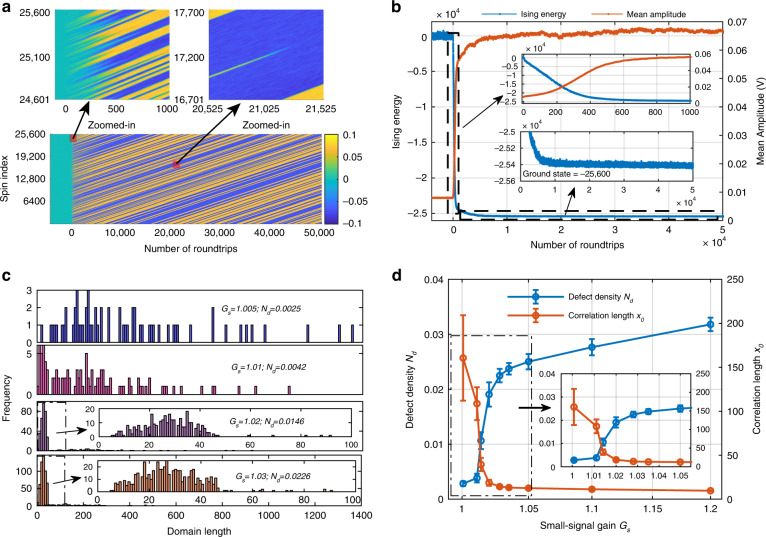
The experimental results of the 1D Ising machine. **a** Spin evolution as a function of the number of roundtrips. The zoomed-in view on the left shows the details of the spin evolution from the noise state to a relatively stable domain. The zoomed-in view on the right shows the evaporation of the short domain. **b** The Ising energy and the mean amplitude of the spins as functions of the number of roundtrips. **c** The histograms of the domain length at different small-signal gains. At the small gain, the domain is relatively long, while the frequency of the shot domain increases at a large gain. **d** The defect density and correlation length as functions of the small-signal gain

Figure 3a, b present the evolutions of the spin and Ising energy as a function of the number of roundtrips in the positive-coupling 1D chain. In the beginning, the oscillation network was in the noise state, and the spins had a small amplitude and uncertain phase, which led to a random global phase configuration. As the number of roundtrips increased, the domains began to emerge, and the domain walls became clear. Simultaneously, some short domains were born and soon swallowed up by the long domains. As the evolution progressed, even some relatively long domains shrank and disappeared, while others grew correspondingly. The longer domains usually had a longer lifetime, and it was difficult for some of them to be destroyed. Ultimately, relatively stable domains were formed. Because the coupling was unidirectional, the domains moved. The speed is linearly dependent on the coupling coefficient (see Section 5 in Supplementary Materials). From the zoomed-in view of Fig. 3b, it can be seen that the mean amplitude of the spins increased very quickly, accompanied by sharp decreases in the Ising energy. The spins took hundreds of roundtrips to reach a relatively stable level, and the machine took the same amount of time to reach low energy, lower than 97% of the ground state.

The ability to reach lower energy is strongly related to the cavity gain^22^. The cavity small-signal gain is changed by tuning the pump current of the Erbium-doped fiber amplifier so that the machine can work at different states. Histograms of the domain length at different small-signal gains *G*_*s*_ = {1.005, 1.01, 1.02, 1.03} are shown in Fig. 3c, from which longer domains can be obtained at smaller values of *G*_*s*_. The defect density, calculated by *n*_*d*_ = *N*_*d*_*/N*, where *N*_*d*_ is the number of domain walls, and *N* = 25,600 is the number of spins, is used to evaluate the performance of reaching lower energy. When the machine operates slightly above the threshold, the defect density can be less than 0.0025. Moreover, the correlation length *x*_0_, which is evaluated by fitting the autocorrelation of the measurement data with $R(x) = 1 - \frac{4}{\pi }{\rm{tan}}^{ - 1}\sqrt {\tanh \left( {\frac{{x^2}}{{4x_0^2}}} \right)}$, is used to further evaluate the machine performance^41^. The relationship between the defect density/correlation length and the small-signal gain is presented in Fig. 3d. This behavior is similar to that in the DOPO-based Ising machine^22^, but the curve is much sharper, which indicates that the proposed machine is much more sensitive to the cavity gain.

**Fig. 4:**
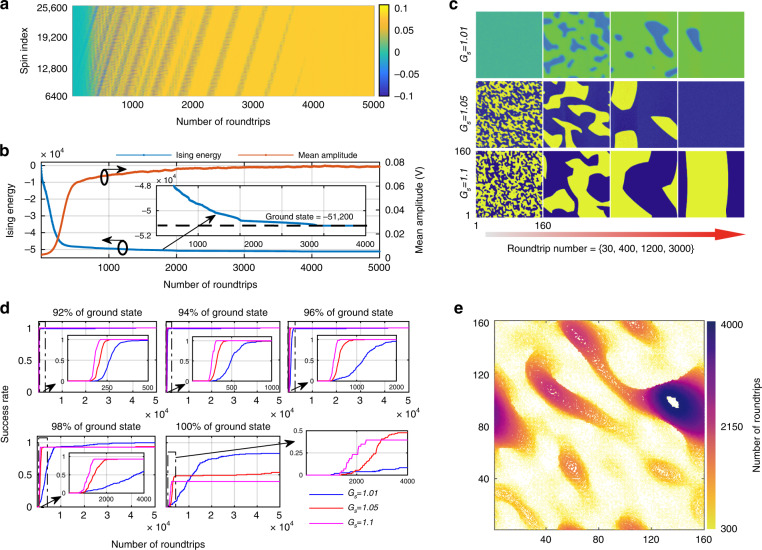
The experimental results of the 2D Ising machine. **a** The spin evolution and Ising energy as a function of the number of roundtrips at *G*_*s*_ = 1.01. **b** The Ising energy and the mean amplitude of the spins as functions of the number of roundtrips. **c** Snapshots of the spin evolution at some specific roundtrips (30, 400, 1200, 3000) at different values of *G*_*s*_ = {1.01, 1.05, 1.1}. At the small gains, the domain walls were smooth and small domains evaporated or merged into larger ones, ending up with a whole. At large gains, the domain walls were sharp at the beginning. As the number of roundtrips increased, the machine was trapped in local energy minima associated with parallel domain walls. The Ising machine reached the ground state in the first two cases and froze out in the last cases. **d** The success rate of cases as a function of the number of roundtrips at which the given percentage of the ground state energy was reached at different small-signal gains. **e** The superposition of domain walls at different numbers of roundtrips. The domain drift was removed through data processing for a better vision. The evaporation and shrinkage of the domain walls show the dynamics of the machine. The change in color of the contour lines from yellow to purple indicates an increase in the number of round trips

By adding a 160-bit delay to implement vertical coupling in combination with the 1-bit horizontal coupling, a 160 × 160 2D Ising square lattice was simulated (see section 3 in Supplementary Materials). Due to the additional constraints imposed by the vertical coupling, the machine took much less time to evolve from the noise state to the low-energy state compared to the 1D Ising simulation, as shown in Fig. 4a. A much steeper curve is observed in Fig. 4b. The machine took less than 4000 roundtrips to reach the ground state.

As with the 1D simulation, the possibility and the time taken to reach lower energy were highly related to the cavity gain. To reveal the evolution process, some snapshots at specific roundtrips at different small-signal gains *G*_*s*_ = {1.01, 1.05, 1.1} are shown in Fig. 4c. At small cavity gains, the machine took longer to reach relatively low energy or a stable spin format but had a higher possibility of finding the ground state. In contrast, large cavity gains induced domain structures consisting of many small domains with sharp domain walls. Figure 4d presents the success rates of reaching 92% to 100% of the ground state at different oscillating amplitudes. At large cavity gains, the machine required several hundred roundtrips to ensure that 96% of the ground state was found, while thousands of roundtrips were required at small cavity gains.

Despite the theoretical prediction that the ground state can always be reached for regular lattices if the calculation time is long enough, it was observed that the domain would freeze out in the DOPO-based Ising machine^42^. A similar freeze-out effect was also observed for the proposed Ising machine, which prevented the machine from reaching the ground state (see video S1 and section 7 in Supplementary Materials). At small cavity gains, the machine was less likely to fall into the freeze-out state and achieved an approximately 76% success rate of finding the ground state within 50,000 roundtrips, whereas the success rate was less than 50% at large cavity gains. For large cavity gains, the Ising energy quickly decreased until the point of freeze-out was reached. In contrast, for low cavity gains, the energy decreased gradually and ultimately reached closer to the ground state before freeze-out occurred. A larger cavity gain corresponded to a higher probability of freeze-out.

One can find that the dynamics in our machine is very similar to that in a DOPO-based machine^42^. Note that evolution continued at small gains; thus, a higher success rate can be expected over a longer period. The contours that separate the spin-up and spin-down domains at every ten roundtrips are superimposed in Fig. 4e to reveal the evolution of the domain. The contours decreased in number along with a gradual decrease in size and finally vanished. Note that the coupling was unidirectional, therefore, the domains moved, which is similar to the situation of the 1D simulation. In the superimposed contours, the movement speed was set to zero via data processing to obtain a better view.

### Solving max-cut problems

**Fig. 5:**
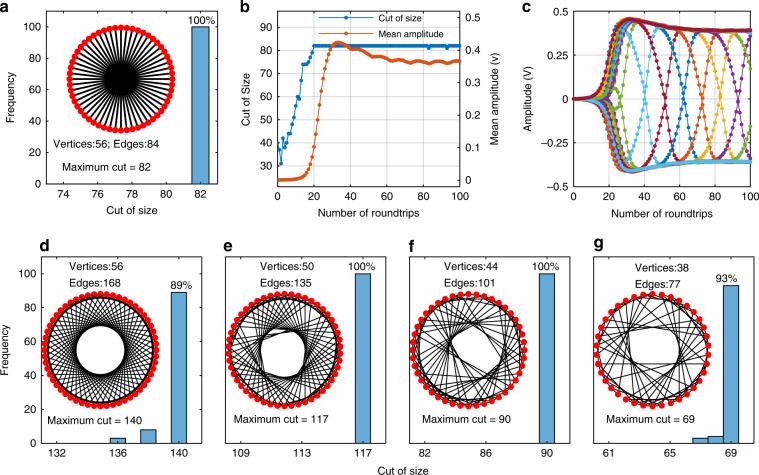
**a** A Möbius ladder graph with *N* = 56 vertices and the histogram of the graph cut of the size that the Ising machine could find in 100 tests. **b** The evolution of the graph cut of size and Ising energy as functions of the number of roundtrips. **c** The spin evolution as a function of the number of roundtrips. **d**–**g** The histograms of cuts of size in different graphs that the Ising machine could find in 100 tests

Max-cut problems are NP-hard problems that can be mapped onto the Ising formulation^11^. The cut of size can be directly mapped by the Ising energy, as follows: $C\left( {\left\{ {\sigma _i} \right\}} \right) = - \frac{1}{2}\mathop {\sum}\nolimits_{1 \le i < j \le N} {J_{ij} - } \frac{1}{2}H\left( {\left\{ {\sigma _i} \right\}} \right)$. For a given ***J***, the lowest Ising energy corresponds to the maximum cut. Here, we programmed unweighted, unidirectional max-cut problems onto the proposed machine. In this demonstration, the 20-km fiber was replaced with a short fiber, in which 56 spins can be supported. First, the maximum cut of a Möbius ladder graph with 56 vertexes was calculated by setting 1-bit and 28-bit delays for the other two channels. The mean amplitudes of the 56 spins as a function of the number of roundtrips are shown in Fig. 5b. In the beginning, the spins were found to have a small amplitude and random phase, which corresponded to a relatively high Ising energy and a small cut of size. As the number of roundtrips increased, the spins evolved toward certain values, either the 0- or *π*-phase with a relatively high amplitude. Meanwhile, the Ising energy decreased while the cut of size increased. At approximately 20 roundtrips, the machine reached the lowest-energy phase configuration, and the given max-cut problem was solved. The computational time is given by *T*_*comp*_ = *N*_*rt*_*T*_*rt*_ = 4.5 μs, where *T*_*rt*_ = 224 ns is the roundtrip time and *N*_*rt*_ = 20 is the number of roundtrips. One hundred tests were performed, and it was found that the machine was able to find the best answer for each test. It should be noted that the coupling was unidirectional, and the amplitude of the spin changed periodically, as presented in Fig. 5c.

Another channel with a 7-bit delay was added to obtain more complex graphics. Different graphics were obtained by programming the output waveform of the arbitrary waveform generator (see video S2 and section 3 in Supplementary Materials). The success probabilities are shown in Fig. 5d–g, from which it is evident that the machine has a relatively high probability of finding the best answer.

## Discussion

The idea of optimization in the proposed Ising machine is similar to that in the DOPO-based Ising machine, i.e., the principle of minimum power dissipation. However, despite the larger number of spins, the proposed machine achieved a lower defect density (0.0025 vs. 0.02)^22^ in 1D Ising model simulation and a higher success rate of finding specific low energy states in 2D Ising model simulation^42^. We believe this performance difference is due to the spin stability. Spin stability, especially in phase, is crucial in those Ising machines that use the relative phase to represent the direction of artificial Ising spins. The phase fluctuation induced by ambient variation or the frequency drift of the local oscillation would change the relative phase and flip the spin, leading to an error computation. The cavity ambient variation and the frequency drift of the local oscillation can be considered cavity noise. Presumably, small noise has a limited effect on the calculation results. However, strong noise will induce random phase flips of the oscillations, similar to temperature-induced spin flips in the Ising model^42^. On the one hand, the noise-induced random spin flips could help the machine escape from local energy minima. On the other hand, it may prevent the machine from reaching a lower energy state, similar to the Ising model with a relatively high temperature. The noise level determines the lower boundary of the effective temperature, which corresponds to the lower energy that can be obtained. Compared to DOPO spins, microwave photonic spins have a longer wavelength, therefore, are less affected by ambient variation. In addition, the local microwave oscillation is usually locked to an atomic clock, which helps to get rid of frequency drift, while the frequency of a laser pump always drifts and is hard to be stabilized. Accordingly, the proposed machine can reach a lower effective temperature than that based on DOPO, and a better performance can be expected.

Another possible reason is that weaker spin-amplitude nonuniformity may be obtained in the OEPO-based machine, leading to a lower Ising energy since it damages the machine performance^43^. Fortunately, the error detection and the feedback control technique used in the DOPO-based Ising machine can also be established in our scheme to improve the machine performance^44,45^. Depending on the comparison between the current energy and the previously visited, one can increase or decrease the cavity gain or the mutual injection strength to help the machine escape from the local energy minima and find a better one. The small signal gain is determined by the power of the pump laser in the OEPO, which can be precisely and independently tuned by an additional intensity modulator.

It should also be noted that the lower Ising energy is obtained at the expense of a slower repetition rate and a longer feedback loop as compared with those of DOPO-based CIMs, which means that a longer time is required to implement a single roundtrip. Fortunately, fewer roundtrips are required to achieve the same low Ising energy in the proposed Ising machine. Moreover, it is worthwhile to obtain lower Ising energy at the expense of a predictable time when solving certain combinatorial optimization problems.

Some platforms have also been proposed to implement Ising machines that can avoid the stability issue of the fiber-based feedback loop of DOPO-based CIMs, e.g., via recurrent feedback using free-space spatial light modulators (SLMs)^27^, the spatial multiplexing of the spins on integrated chips^25,26^, and analog electronic systems^35–38^. Detailed comparisons between different Ising machines are available in section 9 in Supplementary Materials.

Currently, the proposed machine still suffers from connectivity issues. Fortunately, flexible programmability can be easily realized using measurement-feedback schemes, as the microwave spin can be easily measured and controlled with high-speed electrical devices^24^. Taking advantage of its arbitrary programmability, the multi-body interaction and the nonlinear interaction can also be realized. Moreover, recent booming development in photonics matrix multiplication may provide a new way for arbitrary spin interaction^46^. Overall, the microwave photonic Ising machine is expected to be a large-scale, highly-stable, and programable optimizer, thus paving the way for solving real-world combinatorial optimization problems.

## Materials and methods

### Principle of the OEPO

The mixer is the key device required to lock the phase of the oscillating signal with 0 or *π* phase. When the oscillation is degenerate, the transmission function of the mixer can be described as:

1 $$s_{mixer} = \gamma s_{RF} + \beta s_{RF}^ \ast$$

where *β* is the frequency conversion coefficient from the input port, and *γ* is the leakage coefficient of the radio frequency (RF) signal (see section 1 in Supplementary Materials). Assuming the slow-varying envelope applied to the second MZM in Fig. 1a is $s(t) = |s(t)|e^{i\phi (t)}$, the signal after passing through the cavity with delay *τ* can be given as follows^47^:

2 $$s(t + \tau ) = \sqrt {G_{EA}} R_{PD}I_{PD}Z_{PD}J_1\left( {\frac{{\pi \left| {s(t)} \right|}}{{V_\pi }}} \right)\left( {\gamma e^{i\phi (t)} + \beta e^{ - i\phi (t)}} \right)$$

where *t* is the slow time scale of the order of the cavity delay (~100 μs), *G*_*EA*_ is the power gain of the electrical amplifier (EA), *R*_*PD*_ is the responsivity of the PD, *I*_*PD*_ is the power launched into the PD, *Z*_*PD*_ is the PD impedance, *J*_*1*_[·] is the first-order Bessel function of the first kind, and *V*_*π*_ is the half-wave voltage. Near the steady state, *s*(*t*) varies on a slow time scale. The following approximation can be used: $\frac{{ds}}{{dt}}{{{\mathrm{ = }}}}\frac{{s(t + \tau ) - s(t)}}{\tau }$. The dynamic equation is rewritten as:

3 $$\frac{{ds}}{{dt}} = \frac{1}{\tau }\left[ {G_0J_1\left( {\frac{{\pi \left| {s(t)} \right|}}{{V_\pi }}} \right)\left( {\gamma e^{i\phi (t)} + \beta e^{ - i\phi (t)}} \right) - s\left( t \right)} \right]$$

where $G_0 = \sqrt {G_{EA}} R_{PD}I_{PD}Z_{PD}$. Equation (3) can be simplified by extracting the amplitude and phase components:

4 $$\left\{ \begin{array}{l}\frac{{d|s|}}{{dt}} = f_1(\left| s \right|,\phi ) = \frac{1}{\tau }\left[ {G_0J_1\left( {\frac{{\pi \left| s \right|}}{{V_\pi }}} \right)(\gamma + \beta \cos 2\phi ) - |s|} \right]\\ \frac{{d\phi }}{{dt}} = f_2(\left| s \right|,\phi ) = - \frac{{G_0\beta }}{{\tau |s|}}J_1\left( {\frac{{\pi \left| s \right|}}{{V_\pi }}} \right)\sin 2\phi \end{array} \right.$$

When the system is in the steady state, the equilibrium points (|*s*_0_ | , *ϕ*_0_) of Eq. (4) are the solution of *f*_1,2_(*|s* | *, ϕ*) = 0:

5 $$\left\{ \begin{array}{l}\frac{{G_0}}{{|s_0|}}J_1\left( {\frac{{\pi \left| {s_0} \right|}}{{V_\pi }}} \right) = \frac{1}{{\gamma + \beta \cos 2\phi _0}}\\ \phi _0 = k\pi /2,\quad k \in {\Bbb Z}\end{array} \right.$$

Although many solutions |*s*_0_ | satisfy Eq. (5) because the first-order Bessel function is approximately a damped sinusoidal function^48^, only the first/smallest |*s*_0_ | is available in the proposed system. The phase solutions, $\phi _0 = k\pi ,k \in {\mathbb{Z}}$, represents stable points, and $\phi _0 = \pi {{{\mathrm{/2 + }}}}k\pi ,k \in {\mathbb{Z}}$, represents unstable points (see section 1 in Supplementary Materials).

In an OEPO network with *N* oscillations, the interactions between spins can be realized by the injection of the signals of the other OEPOs. Consider *J*_*i,j*_ as the injection coefficient from the *i-*th spin to the *j-*th spin, where $1 \le i,j \le N$. By adding the coupling terms into Eq. (3), the dynamic equation of the *N*-spin OEPO network can be written as:

6 $$\frac{{ds_i}}{{dt}} = \frac{1}{\tau }\left[ {G_0J_1\left( {\frac{{\pi \left| {s_i} \right|}}{{V_\pi }}} \right)\frac{1}{{\left| {s_i} \right|}}\left( {\gamma s_i + \beta s_i^ \ast + \mathop {\sum}\limits_{i < j \le N} {J_{i,j}s_j} } \right) - s_i\left( t \right)} \right]$$

Because the phases of spins are locked at 0 or *π*, all the imaginary parts of $s_i$ are zero, which means $s_i = {\rm{Re}} \left( {s_i} \right)$, and Eq. (6) can be rewritten as a real equation. Without loss of generality, let’s set $\tau = 1$, we can get:

7 $$\frac{{ds_i}}{{dt}} = G_0J_1\left( {\frac{{\pi s}}{{V_\pi }}} \right)\frac{1}{{s_i}}\left[ {\left( {\gamma + \beta } \right)s_i + \mathop {\sum}\limits_{i < j \le N} {J_{i,j}s_j} } \right] - s_i$$

Since the first-order Bessel function of the first kind $J_1[ \cdot ]$ is odd, $J_1\left( {\frac{{\pi \left| {s_i} \right|}}{{V_\pi }}} \right)\frac{1}{{\left| {s_i} \right|}} = J_1\left( {\frac{{\pi s_i}}{{V_\pi }}} \right)\frac{1}{{s_i}}$ is obtained. At the equilibrium, $s_i$ satisfies:

8 $$G_0J_1\left( {\frac{{\pi s_i}}{{V_\pi }}} \right)\frac{1}{{s_i}}\left[ {\left( {\gamma + \beta } \right)s_i + \mathop {\sum}\limits_{i < j \le N} {J_{i,j}s_j} } \right] - s_i = 0$$

When the cavity gain is greater than the threshold, the overall signal decay rate $\Gamma$ can be given as^20^:

9 $$\Gamma = \mathop {\sum }\limits_{i = 1}^N G_0\left( {\gamma + \beta } \right)J_1\left( {\frac{{\pi s_i}}{{V_\pi }}} \right)\frac{1}{{s_i}}$$

According to Eq. (8), we can obtain:

10 $$G_0\left( {\gamma + \beta } \right)J_1\left( {\frac{{\pi s_i}}{{V_\pi }}} \right)\frac{1}{{s_i}} = 1 - G_0\left( {\gamma + \beta } \right)J_1\left( {\frac{{\pi s_i}}{{V_\pi }}} \right)\frac{1}{{s_i}}\mathop {\sum}\limits_{i < j \le N} {J_{i,j}\frac{{s_j}}{{s_i}}}$$

If maximum spin–spin interaction $\mathop {{\max }}\limits_{i < j \le N} \left| {J_{i,j}} \right|$ in the OEPO network is weak enough, the amplitude perturbations of *s*_*i*_ induced by the interactions are small^20^. Ignoring the amplitude perturbations, we assume that all the spin amplitudes are the same. $G_0\left( {\gamma + \beta } \right)J_1\left( {\frac{{\pi s_i}}{{V_\pi }}} \right)\frac{1}{{s_i}}$ is regarded as a constant at the steady state, denoted by $\xi$. Consider $\sigma _i = sign\left( {s_i} \right)$, we have $s_j/s_i \equiv \sigma _i\sigma _j$. Based on Eqs. (5) and (10), the overall signal decay rate is then rewritten as:

11 $$\Gamma = N - \xi \cdot \mathop {\sum}\limits_{1 \le i < j \le N} {J_{i,j}\sigma _i\sigma _j}$$

As one can find that the second term in Eq. (11) has the same form with the Ising model without an external magnetic field. As *N* and $\xi$ are both constants, the signal decay rate *Γ* can be equivalent to the Ising Hamiltonian. Equation (11) conforms to the physical intuition about an oscillator. Assuming each spin has a normalized loss of one in an N-spin oscillation network, the global signal decay rate is N if there is no interaction between those spins. If the *i-*th spin is injected by the *j-*th spin with the coupling coefficient of *J*_*i,j*_, the signal decay rate of the *i-*th spin would rise (if *J*_*i,j*_*σ*_*i*_*σ*_*j*_ < 0) or fall (if *J*_*i,j*_*σ*_*i*_*σ*_*j*_ > 0) by *|J*_*i,j*_ | . Since all the couplings from other spins have an integrated contribution $- \mathop {\sum}\nolimits_{1 \le j \le N} {J_{i,j}\sigma _i\sigma _j}$, the loss of the *i*-th spin is given by $\Gamma _i = 1 - \xi \cdot \mathop {\sum}\nolimits_{1 \le j \le N} {J_{i,j}\sigma _i\sigma _j}$. Accordingly, the global loss of the oscillation network can be expressed as Eq. (11). To solve the Ising problem with the given matrix ***J***, the interactions between the spins are firstly programmed into the oscillation network, either in the analog or in the digital domain. And then, the cavity gain is increased gradually to search for the minimum-loss state. Meanwhile, the amplitudes of spins increase, and their phases are adjusted towards the lowest configuration. When the oscillation network runs stably, one can measure the corresponding phase configuration, from which the best answer to the given problems can be solved.

However, the assumption that all the spins have the same amplitude is not always satisfied. When solving complex problems, the spins experience different interactions along the optoelectronic cavity, which would change the spin gain and ultimately results in amplitude non-uniformity. This non-uniformity may cause inconsistency between the minimal-loss configuration and the optimal solution to the corresponding problem. The negative impact of nonuniformity also can be found in the DOPO-based Ising machine^49^. Fortunately, limiting the amplitude of spins at the feedback and optimizing the nonlinear function could reduce the amplitude inhomogeneity to improve the success probability^43,44^. Details about how the nonuniformity impedes the machine from finding the best answer and how to relieve such negative impact are under investigation.

## Supplementary information

Supplementary Materials

A typical frezee-out evolution

The construction of max-cut solvers
