# Supplementary material for: Large-scale coherent Ising machine based on optoelectronic parametric oscillator
Source: Light Sci Appl. 2022 Nov 25;11:333. doi: 10.1038/s41377-022-01013-1 (PMC9700853; doi:10.1038/s41377-022-01013-1)
Supplement: Supplementary file 1 — Supplementary Materials [file 41377_2022_1013_MOESM1_ESM.docx]

**Supplementary Materials for**

**Large-scale Coherent Ising Machine Based on Optoelectronic Parametric Oscillator**

Qizhuang Cen^1, 2, 3, †^, Hao Ding^4, †^, Tengfei Hao^1, 2, 3, †^, Shanhong Guan^4^, Zhiqiang Qin^4^, Jiaming Lyu^5^, Wei Li^1, 2, 3^, Ninghua Zhu^1, 2, 3^, Kun Xu^4^, Yitang Dai^4, 6^, *, Ming Li^1, 2, 3^, *.

^1^State Key Laboratory on Integrated Optoelectronics, Institute of Semiconductors, Chinese Academy of Sciences, Beijing 100083, China.

^2^School of Electronic, Electrical and Communication Engineering, University of Chinese Academy of Sciences, Beijing 100049, China.

^3^Center of Materials Science and Optoelectronics Engineering, University of Chinese Academy of Sciences, Beijing 100190, China.

^4^State Key Laboratory of Information Photonics and Optical Communications, Beijing University of Posts and Telecommunications, Beijing 100876, China.

^5^School of Optical-Electrical and Computer Engineering, University of Shanghai for Science and Technology, Shanghai 200093, China

^6^Peng Cheng Laboratory, Shenzhen 518052, China.

^†^These authors contributed equally to this work.

^*^Corresponding authors: [ytdai@bupt.edu.cn](mailto:ytdai@bupt.edu.cn); [ml@semi.ac.cn](mailto:ml@semi.ac.cn).

**Content**

[1. The principle of the microwave photonic Ising machine 2](#_Toc102750532)

[1.1 Phase conjugation operation 2](#_Toc102750533)

[1.2 Stability of the equilibrium points 3](#_Toc102750534)

[1.3 Simulations of the OEPO-based Ising machine 5](#_Toc102750535)

[2. Experimental details 6](#_Toc102750536)

[3. Constructions of the Ising model simulations and max-cut problem solver 10](#_Toc102750537)

[4. Random oscillation 12](#_Toc102750538)

[5. 1D Ising model simulation 14](#_Toc102750539)

[6. 1D frustrated Ising model simulation 16](#_Toc102750540)

[7. 2D Ising model simulation 18](#_Toc102750541)

[8. Max-cut problem solver 20](#_Toc102750542)

[9. Comparison and discussion 22](#_Toc102750543)

[10. References and notes 25](#_Toc102750544)

# ****The principle of the microwave photonic Ising machine****

## Phase conjugation operation

In a basic optoelectronic cavity, the microwave signal is up-converted to the optical domain through an optoelectronic modulator, propagates in a long fiber, and is recovered by a photodetector (PD), is then amplified and filtered, and is finally fed back to the modulator. In the proposed Ising machine, the microwave signal passes through an optoelectronic cavity similar to that in the basic optoelectronic oscillator (OEO), while interacting with a local microwave oscillation (LO1) through a second-order nonlinear electronic device, i.e., an electric mixer, as shown in Fig. S1. The second-order parametric process produces the sum frequency and difference frequency of the two input signals. In the proposed design, the difference frequency signal is preserved and serves as the artificial spin, while the sum frequency signal is blocked by a microwave bandpass filter (BPF). As a result, the difference frequency signal at the mixer output, noted as the intermediate frequency (IF) signal, can be given as

$s_{IF}e^{i\omega_{IF}t}\propto s_{LO}e^{i\omega_{LO}t}\left(s_{RF}e^{i\omega_{RF}t}\right)^{*}$,

where $s_{IF}$, $s_{LO}$, and $s_{RF}$ are respectively the complex envelopes of the signals of IF, LO, and RF ports, $\omega_{IF}$, $\omega_{LO}$, and $\omega_{RF}$ are respectively the corresponding angular frequencies, and $\omega_{LO}>\omega_{RF}$.

If the central frequency and the bandwidth of the microwave filter are purposely designed, the IF and RF signals, namely the signals before and after the frequency conversion, share the same frequency. This leads to $\omega_{RF}\text{=}\omega_{IF}\text{=}\frac{1}{2}\omega_{LO}$, after which $s_{IF}\propto s_{LO}s_{RF}^{*}$ can be obtained. Without a loss of generality, assuming the complex envelope of the LO is $s_{LO}\text{=}1$, $s_{IF}\propto s_{RF}^{*}$ is obtained. As is evident, the frequency conversion is degenerate, and it is a phase conjugate operation that reverses the phase of the input signal $s_{RF}$. However, in an imperfect mixer, the input signal would roughly transfer to the output port and mix with the frequency-converted signal. The transmission function of the mixer in the proposed scheme is ultimately given as

$$s_{mixer}=\gamma s_{RF}+\beta s_{RF}^{*},$$

where $\gamma$ is the leakage coefficient of the RF signal from the input port to the output port and $\beta$ is the frequency conversion coefficient from the RF signal to the IF signal. The typical power ratio between the leakage RF signal $\gamma s_{RF}$ and the IF signal $\beta s_{RF}^{*}$ in a commercial electric mixer is between −10 and −30 dB. In this case, the power loss of the signal passing through the mixer depends on the phase difference between the frequency-converted signal $\beta s_{RF}^{*}$ and the leakage signal $\gamma s_{RF}$. The minimum loss is realized when the two parts have the same phase, which means the conjugate of the signal has the same phase as itself, i.e., $s_{RF}^{*}=s_{RF}$. This phase conjugate results in a phase-locking in either 0 or π. Ultimately, the intracavity signal will oscillate with binary phases. The binary phase oscillation then can be used to represent the artificial spin of the Ising model. It should be noted that the frequency conversion in the electric mixer is unconditional and independent of the phase relationship between the two input signals, while the parametric amplification in the DOPO is phase-sensitive.


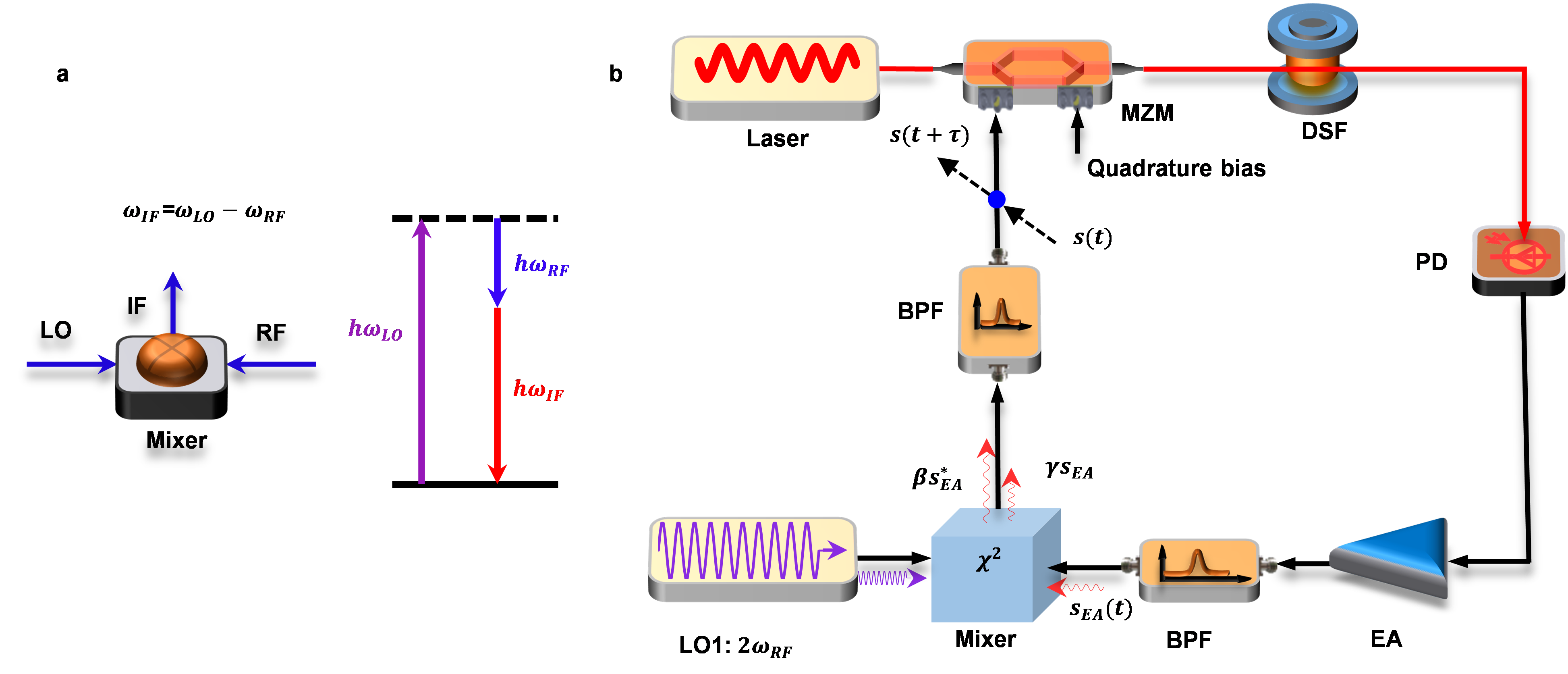


1. The principle of the optoelectronic parametric oscillator (OEPO). **(a)** The schematic diagram and parametric frequency conversion process of the electric mixer. **(b)** The simplified schematic diagram of the OEPO. PD: Photodetector; BPF: bandpass filter; EA: electrical amplifier; MZM: Mach-Zander modulator. DSF: Dispersion-shifted fiber.

## Stability of the equilibrium points

Since the first-order Bessel function is approximately a damped sinusoidal function, equation (5) in the method section has many solutions $\left|s_{0}\right|$^1^. Because the oscillation starts from small noise, which usually includes thermal noise and shot noise, the signal $|s|$ first reaches the smallest/first equilibrium point from zero when the gain is larger than the threshold. Because the cavity gain is limited and can be easily controlled, other equilibrium points are ignored in the subsequent discussion. Here, the Jacobian matrix of the nonlinear differential equation is used to analyze the stability of the equilibrium points. Assuming the smallest solution of Eq. (5) is , the Jacobian matrix of Eq. (4) is given as follows:

The eigenvalues of the matrix are respectively $\left.\kappa_{1}\text{=}\frac{\partial f_{1}}{\partial|s|}\right|_{|s_{0}|}$ and . For a sufficiently small input signal ($|s|\ll V_{\pi}$), the Taylor expansion of the first-order Bessel function is given as follows:

Based on Eq. , the following can be obtained:

.

Because $\left|s\right|\in\left(0,+\infty\right)$ and $\left|s_{0}\right|$ is the first equilibrium point of Eq. (4) in the method section, and have the same sign. If $\frac{\pi G_{0}}{2V_{\pi}}(\gamma+\beta)-1>0$, for , the following can be obtained: , so that ; this means that $\left.\kappa_{1}\text{=}\frac{\partial f_{1}}{\partial|s|}\right|_{\left|s_{0}\right|}\leq0$. Moreover, consider that $\beta>\gamma$, for , the following can be obtained: , , and $\left.\kappa_{2}\text{=}\frac{\partial f_{1}}{\partial|s|}\right|_{\left|s_{0}\right|}\geq0$. The equilibrium point is stable if all eigenvalues have negative real parts, and it is unstable if at least one eigenvalue has a positive real part^2^. As is evident, the eigenvalue $\kappa_{2}$is always less than zero because $\beta>\gamma$. Thus, represents stable solutions, and represents unstable solutions. This means that the oscillation phase will be stably locked to the input signal with two certain phases with a relative phase of $\pi$. The binary-phase stable oscillation then can be used to simulate the spin of the Ising model, and each OEPO is regarded as an Ising spin.

In Eq. (S5), is the cavity gain when the signal is small. The small-signal gain reaches the maximum value $\frac{\pi G_{0}}{2V_{\pi}}(\gamma+\beta)$ when , which means that the cavity selects the signal with specific phases. From this perspective, the oscillation naturally oscillates at relative 0 or $\pi$ phases.

## Simulations of the OEPO-based Ising machine

Here, the noise part was added to Eq. (3), and a simulation was run with MATLAB to obtain the output of the OEPOs. The cavity parameters were modeled as follows: $G_{EA}=30$ dB*,* $R_{PD}=0.8$ A W^-1^/W*,* $I_{PD}=5$ dBm*, $Z_{PD}=50 \Omega$,* $V_{\pi}=\pi$ volts*, $\beta=0.35$,* $\gamma=0.05$, $\tau=100 \mu s$, and the noise floor was modeled as Gaussian white noise with a power spectral density of −150 dBm Hz^-1^.

Twenty oscillations were simulated, each representing a single artificial spin of the Ising model. The amplitude of the spins is shown in Fig. S2a, from which we can see that the oscillation amplitude reached the maximum value and remained stable after about dozens of roundtrips. Unlike the monotonic amplitude increase, the spin phase fluctuated around some specific values at the initial roundtrips due to the phase conjugation operator, as shown in Fig. S2b. Furthermore, the wobbles became smaller with the increase in roundtrips, and the spin phases finally converged to 0 or π. The signal began from noise, and each spin had an equal probability of evolving to 0 or π at the steady state without coupling. The stability of the oscillation was evaluated by calculating $\frac{|s(t+\tau)-s(t)|}{|s(t)|}$. The results are shown in Fig. S2c, from which it can be concluded that the spin was highly stable.

As presented in Fig. S2d and S2e, the evolution of two coupled OEPOs was simulated. Based on Eq. (4), the possible steady state of the system depends on the value $\left\{J_{i,j}\right\}$. The phase diagrams of the two coupled OEPOs in Fig. S2d and S2e reveal how the spins evolved to specific states. The arrows indicate that all initial phase states eventually converged to these stable states, as indicated by red crosses. Figure S2d presents the phase diagram with positive spin coupling ($J_{1,2}>0$); as expected, the spin was found to tend to evolve toward the same value, corresponding to the 1D ferromagnetic Ising model. When the spin coupling $J_{1,2}$ was negative, the spin tended to evolve toward the opposite value and corresponded to the antiferromagnetic 1D Ising model, as shown in Fig. S2e.


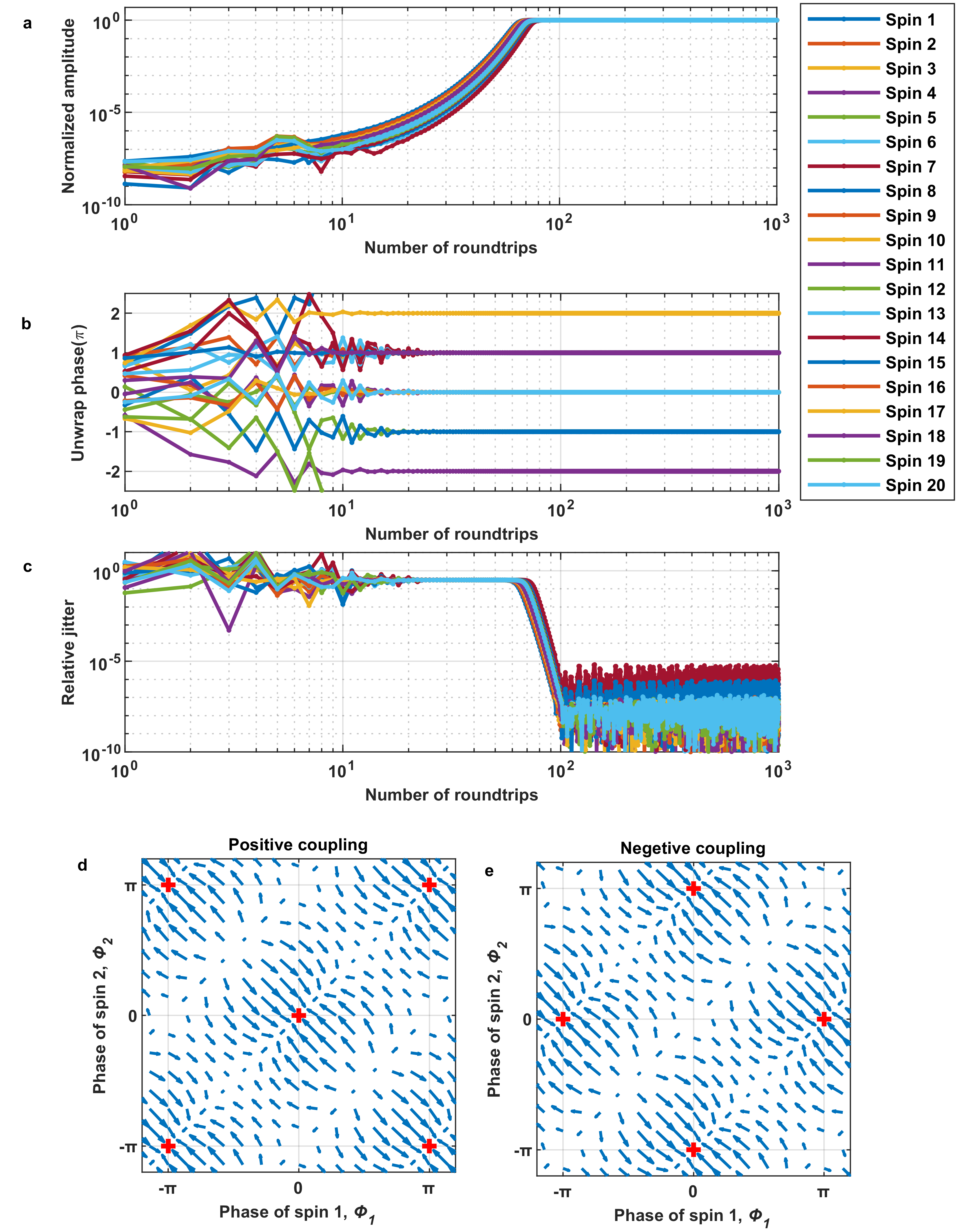


1. The simulation of the parametric microwave photonic spin. **(a-c)** The evolution of the spin amplitude **(a),** the phase **(b),** and the relative jitter **(c)** as a function of the number of roundtrips. **(d, e)** The phase diagrams of two coupled OEPOs with **(d)** $J_{1,2}>0$ and **(e)** $J_{1,2}<0$.

# ****Experimental details****

The experimental setup is shown in Fig S3. Continuous light waves with a 100-GHz frequency space and given wavelengths from 1549.32 to 1551.72 nm were provided by a multi-channel laser (IDPHOTONICS, CoBrite_DX4_). These light waves were coupled through a wavelength division multiplexer (WDM) and shaped into pulse trains via an electrical pulse train and a low-bias intensity modulator. An electrical pulse with a repetition of 250 MHz and a 20% duty cycle was generated from an arbitrary waveform generator (Tektronix AWG70001A). The shaped optical pulse train was then used as the carrier of the microwave signal. To minimize the dispersion, a 20-km DSF was used to store the spins. The PD (DSC40S) in the experiment had a 3-dB bandwidth of 18 GHz and a responsivity of 0.75 A W^-1^ at 1550 nm.

**Frequency conversion:** The frequency conversion device in the proposed OEPO-based Ising machine is an electric mixer (Marki M2-0020)^3^, the size of which is about $50\times30\times10$ mm. In the proposed Ising machine, the 10-GHz oscillating signal (RF) and the 20-GHz local oscillation (LO1) are launched into these two input ports. The IF output is the frequency-converted signal with a frequency of 10 GHz, the same as that of the RF signal. This frequency conversion process is similar to that of the phase-sensitive amplifiers (PSAs) in the DOPO-based Ising machines and is a degenerate process. Due to the degenerate process, binary phase oscillation can be achieved in either the OEPO or the DOPO, which is the key to implementing an Ising machine.

**Degenerate oscillation in the OEPO:** After photodetection, the signal is filtered by an 8-12 GHz BPF and amplified, and is then divided into two parts: one part is used for measurement, and the other is launched into an electric mixer and frequency-converted by LO1. A second 8-12 GHz BPF is used to suppress the sum-frequency and residual LO1 signals. The filtered signal is then amplified and fed back to a quadrature-biased MZM. If the width of the pulse train and the bandwidth of the BPF are carefully designed, the degenerate oscillation can be obtained by setting the correct frequency of LO1. The LO1 frequency $f_{LO1}$, the oscillating microwave frequency $f_{s}$, and the FSR of the optoelectronic cavity $f_{FSR}$ satisfy $f_{LO1}={2f}_{s}=2Mf_{FSR}$, where *M* is an integer. The microwave signal oscillates from the noise state, which provides a random phase if no spin-spin interaction occurs, and then increases in amplitude while the phase converges to either 0 or *π*.

**WDM system:** The WDM system is used to implement spin-spin interaction. In the optoelectronic cavity, a multiplexer/demultiplexer pair is adopted to enable the independent delay operation of each channel. Four tunable optical delay lines (ODLs) are inserted between the multiplier and the demultiplexer. Spins carried by different channels pass through the different paths with specific delays. When the signals of different channels are combined and detected in the PD, the interaction is realized. Lightwave interference between different channels is avoided, as the neighboring channel interval (100 GHz) is much larger than the bandwidth of the optical pulses (about 1 GHz). For example, the WDM system with two channels with a 1-bit delay difference causes interaction between the (*i-*1)-th spin and the *i*-th spin. By tuning the laser switches, the generation of a random microwave pulse train or the simulation of the Ising model can be realized. In the random microwave pulse train generation, only one channel is on. In the simulation of the 1D/2D Ising model, two or three channels are used. The coupling coefficient, either the strength or the sign, is realized by tuning the laser power or the ODLs. Because the spin frequency is about 10 GHz, according to a period of 100 ps, ODLs with a 500-ps tunable range can easily realize the positive or negative coupling. Note that the optical frequency or phase plays no role in the transmission of spins in the WDM channels. The 100-GHz frequency spacing of the WDM ensures that the different optical carriers in these channels have no contribution to the spin-spin interaction. Since the spin is represented by the microwave phase with 10-GHz frequency, any channel spacing significantly larger than 10 GHz is acceptable.

**Cavity length control:** The fiber is placed in a self-fabricated thermostat to suppress the long-term instability caused by the temperature sensitivity of optical fiber. The temperature fluctuation in the thermostat is about ±0.2 °C, which means that the variation of delay can be as large as 136 ps in a 20-km fiber. A phase-locked loop is used to further stabilize the cavity delay, as shown in Fig. 3S. A continuous-wave (CW) laser with a wavelength of $\lambda_{5}=1552.52 \mathrm{nm}$ is used to carry a radio frequency signal with 2.5 GHz, which is locked to LO1. The 2.5-GHz signal carried by $\lambda_{5}$ is combined with other channels through the optical coupler, passes through the long fiber to sense the change in the length of the cavity induced by the temperature fluctuation, and is then coupled through the demultiplexer and recovered by the PD. If the link delay is unstable, the phase of the recovered 2.5-GHz signal changes accordingly. A phase detector is used to obtain the phase jitter, after which the link delay is compensated by a proportional integral derivative (PID) unit and a tunable ODL. The tuning range of the ODL is 500 ps, which is sufficient to compensate for the link jitter.

**Measurement:** To extract the phase states of the oscillating microwave pulses, another external microwave (LO2), synchronized to LO1 with a fixed frequency of $f_{LO1}/2=10$ GHz and a power of 10 dBm, is used to demodulate the oscillating signal. The demodulated baseband signal passes through a low-pass filter (LPF) with a 3-dB bandwidth of 1 GHz and is then divided into two parts: one part is launched into the high-speed, real-time oscilloscope (Tektronix, DPO70000), and the other is launched into an extra digitizer with a 700-MHz analog bandwidth (Teledyne ADQ12). The digitizer is synchronized to the electrical pulse and samples the demodulated baseband signal with the same frequency. The demonstrated baseband signal is a pulse train with positive or negative polarity, which indicates the 0 or *π* phase relative to LO2.

**Relevant channels in the frequency domain:** A sketch map of the relevant channels in different cavity positions of the proposed Ising machine in the frequency domain is shown in Fig. S3b. The different optical carriers from the multi-channel laser are combined at position ① by using the WDM as a beam adder. The combined CW optical signal is then shaped into optical pulses by the 250-MHz electrical pulses at the first modulator. The oscillating microwave signal centered at the 10-GHz frequency is loaded onto the optical pulses at the second modulator. After spin-spin interaction, the optical pulses are converted into microwave signals at the PD. Finally, the microwave signals are filtered, frequency-converted, and fed back to the second modulator to form a closed OEPO loop.





1. **(a)** The experimental setup. MUX: multiplexer; DEMUX: demultiplexer; QB: quadrature bias; ESA: electrical spectrum analyzer; ADC: analog-to-digital converter. **(b)** The sketch map of relevant channels in the frequency domain in different cavity positions of the proposed Ising machine. ①: the optical spectrum of the four-channel CW laser. Laser frequencies are aligned to the center of the corresponding channels of the MUX. ②: the optical spectrum of the modulated optical pulses. ③: the optical spectrum of the optical pulses modulated by 10-GHz microwave pulses. ④: the spectrum after the PD. ⑤⑧: the spectrum of 10-GHz microwave pulses after the BPF. ⑥: the spectrum of the microwave at the mixer output. ⑦: the spectrum of the local oscillation 1. ⑨: the spectrum of the demodulated baseband pulses.

# ****Constructions of the Ising model simulations and max-cut problem solver****

**1D Ising model simulation:** In the 1D simulation, as shown on the left side of Fig. S4a, two channels were used to carry the same microwave signal and were passed through two paths, which had a 1-bit delay difference. The short path was used as the signal path, and the long path was used as the coupling path. As a result, the (*i-*1*)*-th spin was coupled into the *i*-th spin, with a boundary condition of the 25,600-th spin to the first spin. It should be noted that the coupling was unidirectional, which means that *J_i-1,i_*≠0, while *J_i,i-1_*=0. The simulated 1D Ising model was a closed loop with 25,600 nodes, as shown on the right side of Fig. S4a.

**1D frustrated Ising model simulation:** An additional channel with a 2-bit delay was supplemented with a phase of $\pi$ for the 1D frustrated Ising chain, which is shown on the left side of Fig. S4b. By doing so, the *i*-th spin was injected by the (*i*−1)-th and (*i−*2)-th spins. The additional 2-bit coupling caused an opposite spin among the next-nearest neighbors and was in conflict with the 1-bit channel; thus, frustration occurred.

**2D Ising model simulation:** In the 2D Ising model simulation, an additional channel was used to implement vertical coupling based on the 1D simulation. This coupling path had an extra 160-bit delay compared to the signal path. As a result, the *i*-th spin was injected by the (*i*−1)-th and (*i−*160)-th spins, with boundary conditions of the 25,600-th spin to the first spin and the (25,440*+k*)-th spin to the *k*-th spin, where 1 ≤ *k* ≤ 160. Accordingly, the 25,600 spins are assembled as a 160×160 square lattice, as shown in Fig. S4c.

**Solving max-cut problems:** Different max-cut graphs can be implemented by properly designing the temporal sequence of the Ising spins, even if the cavity delay and coupling channels are both fixed. For example, a max-cut graph with 10 vertices can be obtained with a four-channel WDM system with additional 1-bit, 3-bit, and 5-bit delays, as shown in Fig. S4d. By properly designing the temporal sequence of the Ising spins, as shown on the left side of Fig. S4e, a different max-cut graph with six vertices and special connectivity was obtained. In the experiment, the specific temporal sequence of the Ising spins was realized by programming the output of the electrical arbitrary waveform generator (AWG). For a more intuitive representation, please refer to Video S2.





1. **(a)** The schematic diagram of the 1D Ising simulation. Left: the two-channel WDM system with a 1-bit delay. Right: the 1D Ising graph. **(b)** The schematic diagram of the frustrated Ising model of the 1D Ising chain. Left: the three-channel WDM system with 1-bit delay and 2-bit delay. Right: the Ising graph. The additional 2-bit delay has a phase of $\pi$, which introduces frustration to the 1D Ising chain. **(c)** The schematic diagram of the 2D Ising simulator. Left: the three-channel WDM system with 1-bit delay and 160-bit delay. Right: the 2D Ising graph. **(d, e)** An illustration of the implementation of different max-cut graphs. **(d)** A max-cut graph with 10 vertices, in which a four-channel WDM system with 1-bit, 3-bit, and 5-bit delay is used. The Ising spins have a simple temporal sequence. **(e)** A max-cut graph with six vertices and special connectivity, for which a four-channel WDM system with 1-bit, 3-bit, and 5-bit delay is also used under the same cavity length. The temporal sequence of the Ising spins is precisely designed to obtain the graph.

# ****Random oscillation****

The high stability means the dynamics of the spins are less influenced by ambient perturbation, while mainly by the interactions between the spins. We confirm the high stability of the spins through the no-interaction oscillation network. The oscillation process from noise to stability was recorded and is presented in Fig. S5a. The spins took about 60 roundtrips to reach a stable amplitude and phase. The corresponding power spectrum was also measured by an electrical spectrum analyzer (Rohde & Schwarz FSW43), and the results are exhibited in Fig. S5b. The power spectrum was a microwave frequency comb with frequency spacing equal to the optoelectronic cavity’s free spectrum range (FSR). The clear comb line with a high-resolution bandwidth (RBW) in the zoomed-in view suggests the high stability of the spins. The comb line with FSR spacing also indicates that the oscillation period was the cavity roundtrip time, and the spins within one period oscillated randomly. The autocorrelation was calculated, as presented in Fig. S5d. Only one peak was observed within the whole span, indicating that each oscillation was independent and was randomly either the 0 or *π* phase. The cross-correlation between two tests was also calculated. For each test, the machine restarted from noise. No peak was observed along the entire span in the cross-correlation, which indicates that each test was independent.

Over two million traces were recorded in about 12 hours by using a high-speed oscilloscope under the persistence mode, and the results are shown in Fig. S5f. The open-eye waveform indicates that the spins were highly stable both in amplitude and phase; the “eye” would be closed in an unstable system, as its amplitude and/or phase would vary over time.





1. **(a)** The oscillation process from the noise state to the stable state. **(b)** The 6-GHz span power spectrum in the 10-kHz RBW. **(c)** The details of the power spectrum in the 10-Hz RBW. **(d)** The comparison between autocorrelation and cross-correlation. The correlation was calculated using the peak value of the demodulated pulses measured by the ADC. **(e)** The zoomed-in views of the correlations in (d). **(f)** The overlay of 178,631 traces of the demodulated baseband of the OEPO in about 1 hour. **(g)** The overlay of 2,013,944 traces of the demodulated baseband of the OEPO in about 12 hours.

# ****1D Ising model simulation****

The peaks of the demodulated baseband pulses in the 1D positive Ising simulation were recorded by the ADC and are plotted in Fig. S6a. The spin amplitude increased as a function of the small-signal gain, and the domain was relatively long at a small gain. The shape of the domain wall was also found to be strongly related to the cavity gain, as is evident from the zoomed-in view of the waveform. When the machine worked at small amplitude, the transition between domains was gradual, and the spin amplitude was unstable. The amplitude nonuniformity was mainly due to the injection from other spins and, to a lesser extent, the perturbation of cavity noise. As the amplitude increased, the domain walls became steep. The autocorrelation of the experimental data was found to match well with the fitting function $R\left(x\right)=1-\frac{4}{\pi}\tan^{-1}\sqrt{\tanh\left(\frac{x^{2}}{4x_{0}^{2}}\right)}$, where $x_{0}$ is the correlation length.

The shapes of the domain wall at different cavity gains were extracted and normalized, as shown in Fig. S6b. In the proposed machine, the domain drift speed equals the coupling strength $J_{i-1,i}$, where the subscript means that the coupling is from the (*i*-1)-th spin to the *i*-th spin. The length of the domain wall is $\sqrt{J_{i-1,i}\left(1-J_{i-1,i}\right)/\ln G_{s}}$, where the $G_{s}$ is the small-signal gain. Using these parameters, we normalized the domain walls, as shown in the insets of Fig. S6b. One can find that the domain walls at different cavity gains have the same normalized shape. These results are the same as that in the DOPO-based Ising machine^4^.

The net magnetization $\left\langle\left|M\right|\right\rangle$ of the Ising model as the function of the normalized temperature $T_{n}$ presents in Fig. S6c. No phase transition is observed at the finite temperature. The 1D Ising model is from the paramagnetic phase ($\left\langle\left|M\right|\right\rangle=0$) to the ferromagnetic phase ($\left\langle\left|M\right|\right\rangle=1$) near the zero temperature. We believe that the system will evolve to the ferromagnetic phase when $T_{n}=0$.

In a conventional oscillator, the time to stable oscillation depends on the cavity gain. Near the threshold, the oscillation might be unstable or may require a longer amount of time to reach stability; for a larger gain, the oscillation takes much less time to operate stably. The color plots of the demodulated baseband amplitude as functions of the number of roundtrips (horizontal) and the spin index (vertical) at different small-signal gains *G_s_* = {1.005, 1.01, 1.02, 1.03} are exhibited in Fig. S6d. At small cavity gains, the spins required more roundtrips to form stable domains, but a longer domain length could be formed in the beginning. At large cavity gains, only a few hundred roundtrips were required to form stable domains.

Because the coupling was unidirectional, the domains drifted, as can be observed in Fig. S6e. By tuning the laser power of the 1-bit delay channel, the coupling coefficient *J_i-1,i_* can be changed. Theoretically, the speed of the domain drift is equal to the coupling coefficient. The coupling coefficient was tuned from 0.21 to 0.51, and the corresponding drift speeds were calculated. The experimental data agreed with the theoretical calculation^4^, as shown in Fig. S6f.


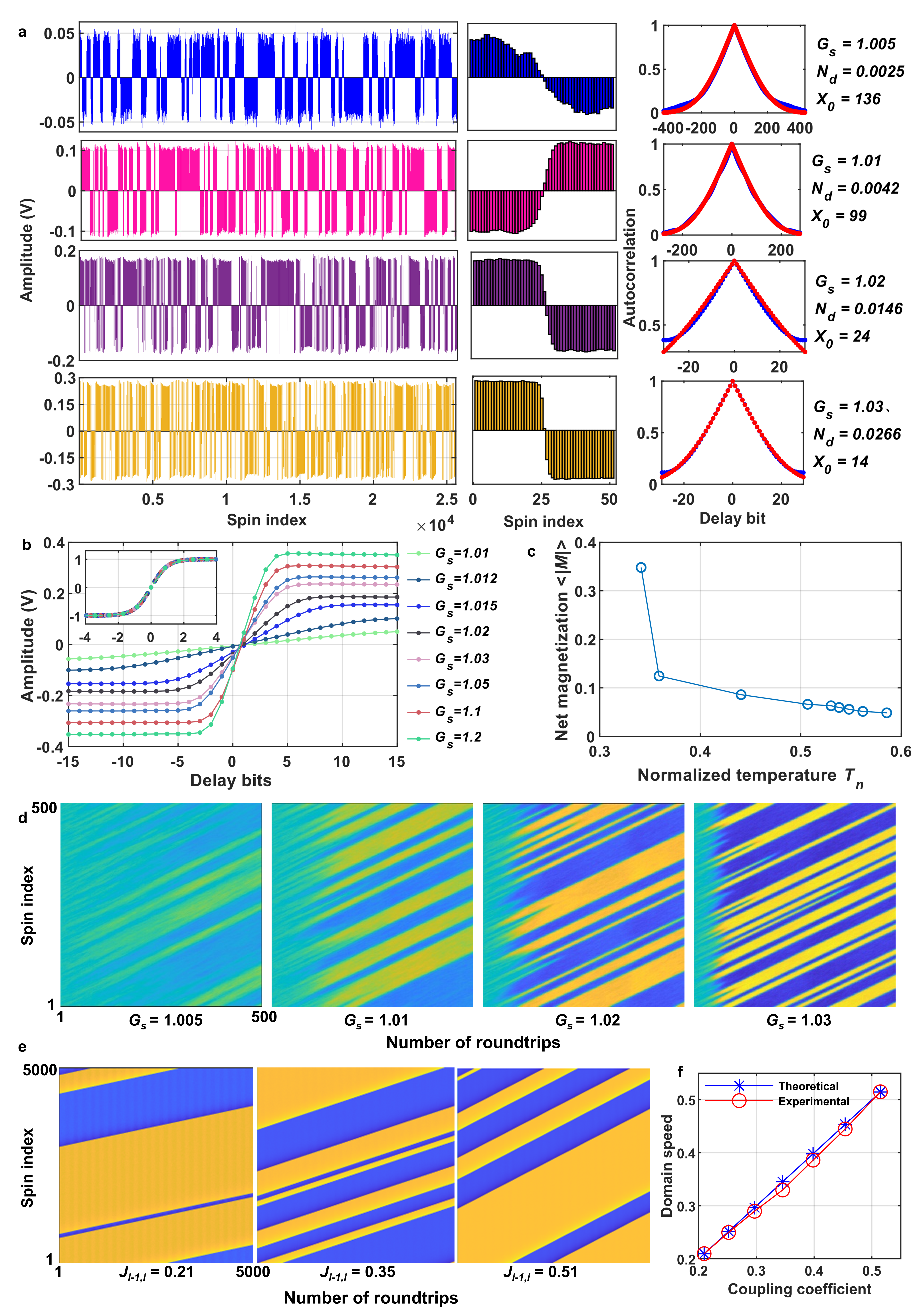


1. **(a)** Left: the demodulated baseband pulse train at different cavity gains. Middle: the zoomed-in view of the left side. Right: the autocorrelation (blue) and the corresponding fitting function (red). **(b)** The normalized domain wall at different small-signal gains. **(c)** The net magnetization as the function of the normalized temperature. **(d)** The spin evolution at different small-signal gains. **(e)** The color plots represent the speed of the domain corresponding to the coupling coefficients {0.21, 0.35, 0.51}. **(f)** The theoretically calculated and experimentally measured speed of the domain.

# ****1D frustrated** **Ising model simulation****

In the current setup, the coupling strength is controlled by the laser power of the corresponding channel, and the tuning of the optical delay line can change the sign of the coupling. As a result, the proposed machine can operate in a frustrated regime. The frustrated 1D chain was implemented by adding another channel with a 2-bit delay and negative coupling. As shown in Fig. S7a, the (*i*-2)-th and the (*i*-1)-th spins were coupled into the *i*-th spin with coupling strengths of *J_i-1,i_* and *J_i-2,i_*, respectively. A frustrated 1D chain in this system can be implemented in two cases, i.e., case I: *J_i-1,i_* > 0, *J_i-2,i_* < 0, and case II: *J_i-1,i_*, *J_i-2,i_* < 0. In both cases, the (*i*-2)-th spin directly opposites the *i*-th spin through the coupling *J_i,i-2_*, while aligning *i*-th through the coupling *J_i,i-1_* and the (*i*-1)-th spin. This conflict causes frustration. Figure S7b presents the transition from antiferromagnetic to ferromagnetic as the coupling parameters *J_i-1,i_*, and *J_i-2,i_* vary. Several typical pairs of parameters in the phase diagram were chosen to reveal the behavior of the 1D frustrated system in the experiment, denoted as black crosses in Fig. 7Sb. The corresponding output demodulated waveforms are presented in Fig. S7c-h. One can find that the waveform output changed from antiferromagnetic to ferromagnetic with the variation of the coupling parameter. This behavior is similar to that in the DOPO-based machine^4^.


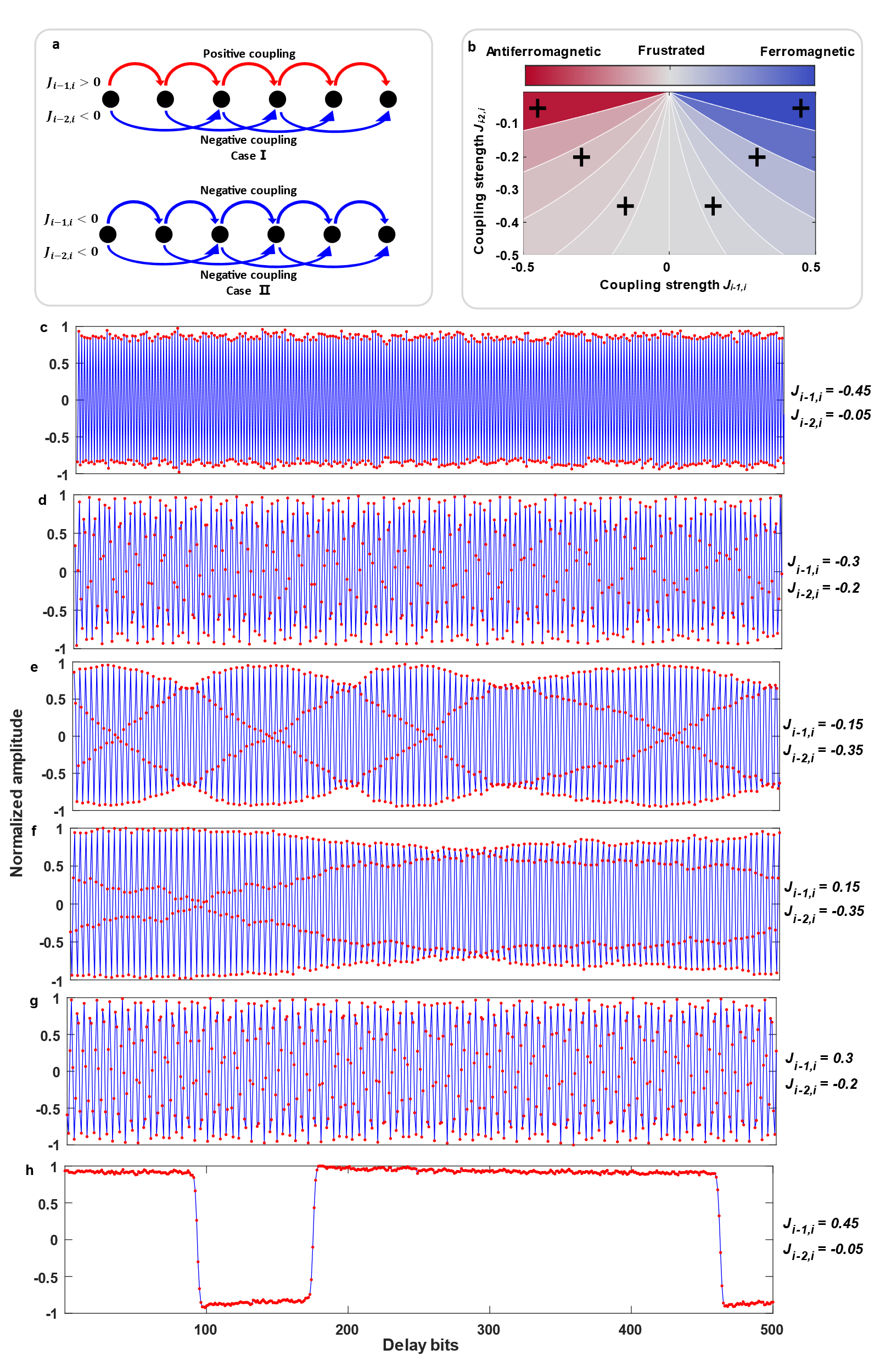


1. **(a)** The couplings for the implementation of the 1D frustrated chain. **(b)** The phase diagram for the frustrated 1D system in the experimental setup. **(c)** The experimental output with the variation of the couplings $J_{i-1,i}$ and $J_{i-2,i}$ in the 1D frustrated system.

# ****2D Ising model simulation****

Similar to the DOPO-based CIM^5^, the proposed Ising machine does not always find the ground state; it sometimes freezes out, especially when the cavity gain is significantly large. The extension of the freeze-out domain can be horizontal, vertical, southwest-northeast diagonal, or northwest-southeast diagonal, as shown in Fig. S8. Interestingly, the machine ultimately froze out in only four types of phase formats in the experiment. In these states, the domains moved, as the couplings in both the vertical and horizontal directions were unidirectional. Several snapshots are presented to reveal the formation of the freeze-out formats. For a typical evolution of the freeze-out 2D Ising model simulation, please refer to Video S1.





1. Four instances of freeze-out in the 2D Ising simulation. **(a)** The Ising energy as a function of the number of roundtrips. **(b-e)** The corresponding snapshots of the spin evolution of the four cases at different roundtrips.

Sometimes, even the freeze-out phase format can be destroyed, as shown in Fig. S9. At around the 5000th roundtrip, horizontal domains were formed, and the Ising energy stopped falling; however, the evolution did not stop. The smaller domain thinned down and tended to be a line. When the line was broken, the Ising energy sharply decreased. The machine reached the ground state in less than hundreds of roundtrips.


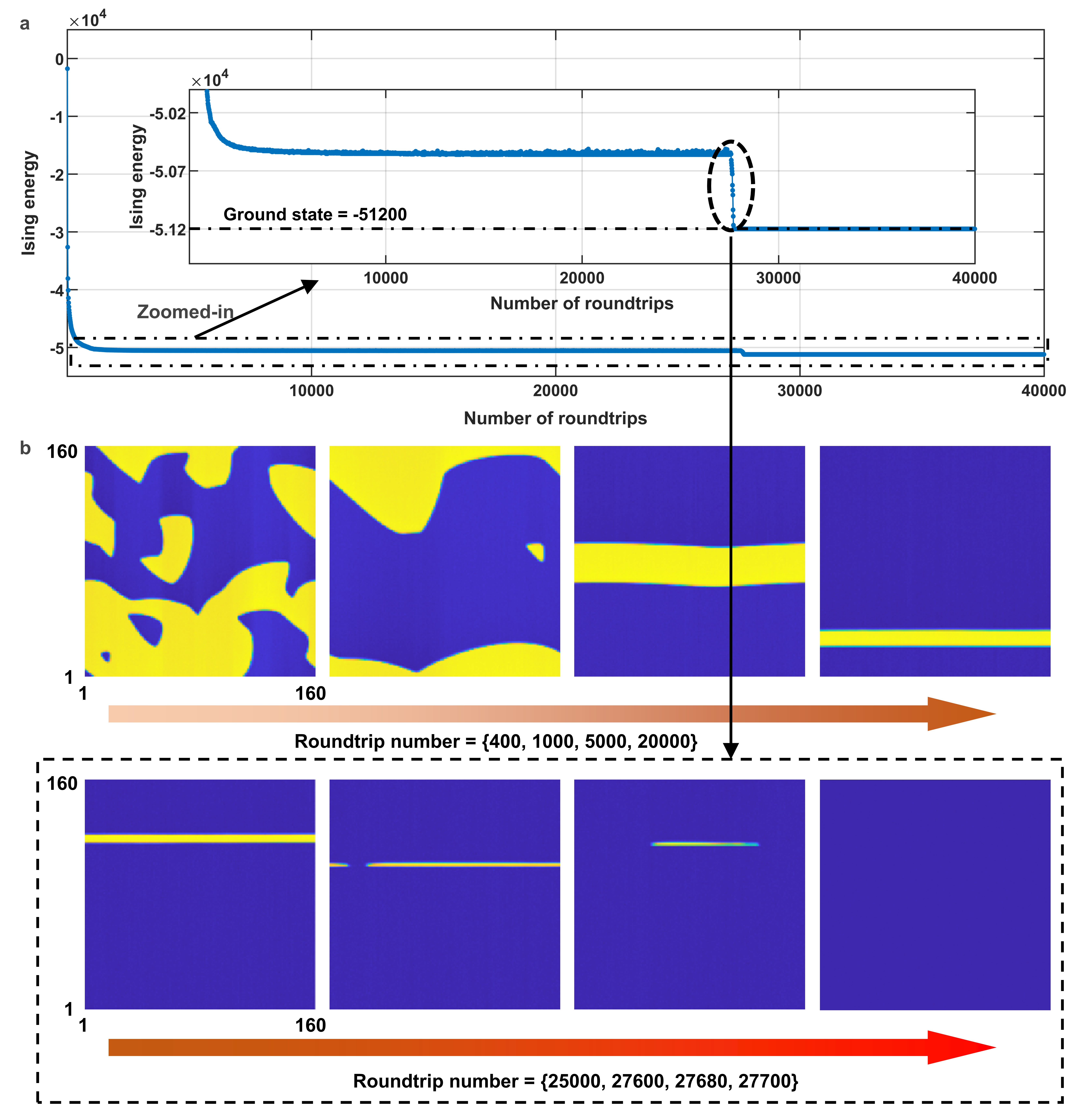


1. The breaking of a freeze-out format in the 2D Ising simulation. **(a)** The Ising energy and the mean amplitude as functions of the number of roundtrips. **(b)** The snapshots of the spin evolution at different roundtrips.

# ****Max-cut problem solver****

By programming the output waveform of the AWG, different graphs can be constructed with a degree of freedom; please refer to Section 3. The absent pulses in the AWG output were randomly chosen by the MATLAB algorithm. The proposed machine was used to find the maximum cut of the corresponding graphs, and the experimental results are displayed in Fig. S10. One hundred tests were performed for each max-cut graph problem. It was found that the machine had a high possibility of finding the maximum cut for most of the graphs. The success rate of finding the best answer is related to the graph structure. The best answer or the maximum cut of the graphs was obtained with the help of the following website: neos-server.org^6^.


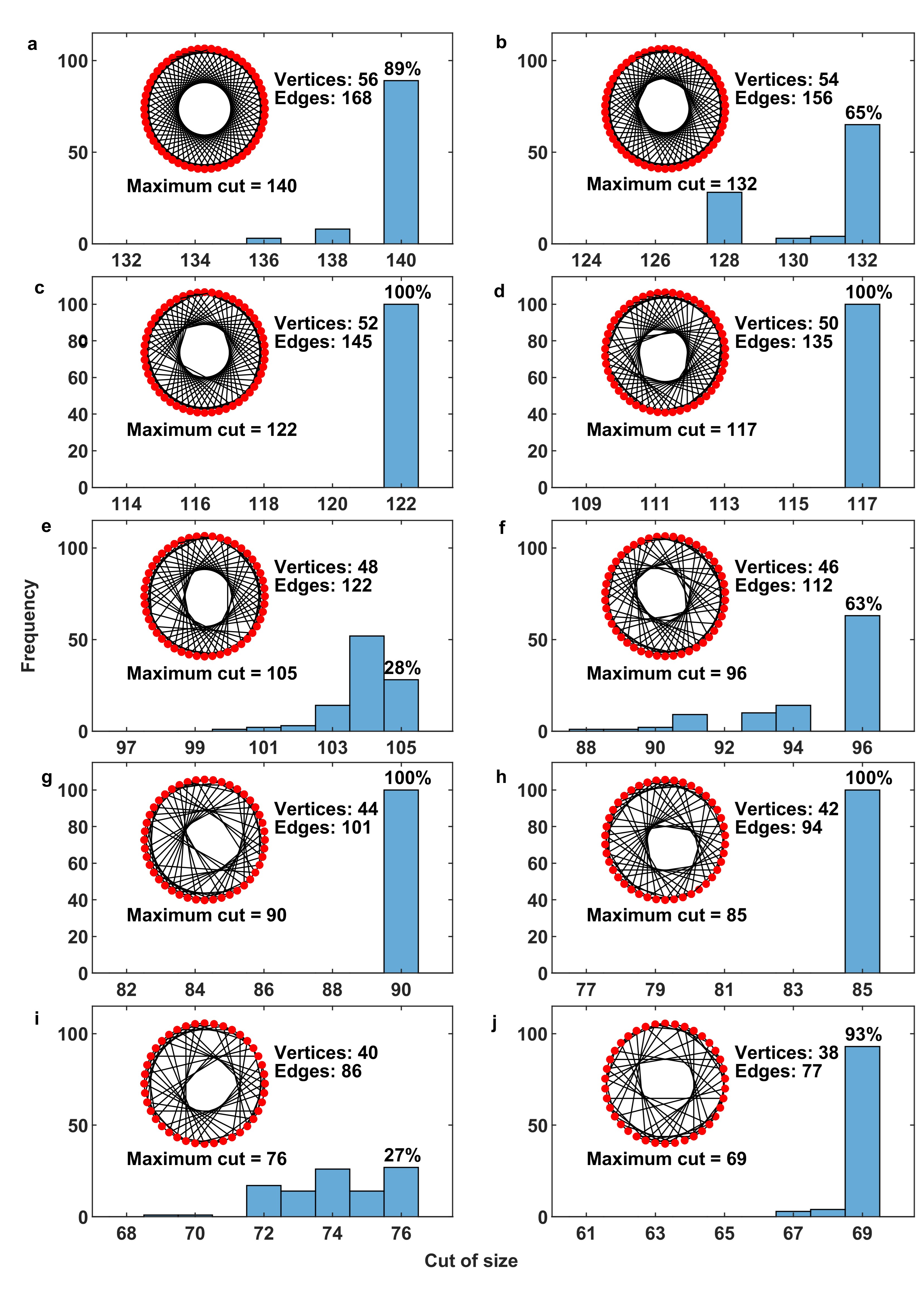


1. The graph structures and the corresponding histograms of the cuts of size that the proposed machine found in 100 tests.

# ****Comparison and discussion****

A quantitative comparison of the proposed OEPO-based CIM and current state-of-the-art Ising machines is given in Tab. S1. The key parameters, such as time to solution, success probability, scale, and operating temperature, are given and compared.

Table S1 Comparison of the proposed CIM and current state-of-the-art Ising machines.

|  | The proposed scheme | DOPO^7–9^ | LC- oscillator^10^ | Super-conducting ^11,12^ | Memristor^13^ | SLM^14^ | Photonic RNN^15^ | SRAM^16^ |
| --- | --- | --- | --- | --- | --- | --- | --- | --- |
| Optimization mechanism | MLP | MLP | MLP | QA | HNN | SA | RNN | SA |
| Scale (*N*)^(1)^ | 2.5×10^4^ | 10^5^ | 240 | 5000 | 60 | 10^6^ | 64 | 6×10^4^ |
| Degrees of freedom of programmability^(2)^ | *N*^2^ | *N*^2^ | *N*×*d*  (*d* = 4) | *N*×*d*  (*d* = 15) | *N*^2^ | 2×*N* | *N*^2^ | *N*×*d*  (*d* = 6) |
| Stability | >12 *h* | ~4 *s* | \ | 76 *μs* | \ | \ | \ | \ |
| Defect density  (1D Ising model) | 0.0025 | 0.02 | \ | \ | \ | \ | \ | \ |
| Time to solution^(3)^ | 22.4 *μs* | 30 *μs* | \ | 10^4^ *s* | 600 *ns* | \ | \ | \ |
| Success probability  ^(4)^ | 100%  (*N* = 56) | 40%  (*N* = 56) | \ | \ | 54%  (*N* = 60) | \ | \ | \ |
| Scaling of success probability for *N*×*N* problems^(5)^ | $e^{-N}$ | $e^{-N}$ | $e^{-N^{2}}$ | $e^{-N^{2}}$ | $e^{-N}$ | $e^{-N}$ | $e^{-N}$ | $e^{-N^{2}}$ |
| Clock frequency | 250 MHz | 5 GHz | 1 MHz | \ | 500 MHz | 0.03 Hz | \ | 100 MHz |
| Temperature | RT | RT | RT | <22 *mK* | RT | RT | RT | RT |

SLM: spatial light modulator; SRAM: static random access memory; ML: minimum loss principle; QA: quantum annealing; HNN: Hopfield neural network; SA: simulated annealing; RNN: recurrent neural network; RT: room temperature. Unavailable quantities are denoted as “\”.

1. The scale of the spin refers to that of physical spin.
2. The degree of freedom of programmability refers to the number of elements in the coupling matrix ***J*** that can be programmed arbitrarily. The proposed machine can be easy to realize *N×N* programmability with the help of the measurement-feedback scheme used in the DOPO-based Ising machine. In LC-oscillator-, superconducting-, SRAM-based Ising machines, the scale of the logical spin is much less than that of the physical spin in solving the fully-connected problems since the spins are locally connected. For a fully-connected model, *N* physical spins can only support about $\sqrt{N\times d}$ logical spins, where *d* is the available degree (the number of allowed couplers) of a physical spin^17^. The SLM can be used for arbitrary programming in the case of scale reduction from *N* to $\sqrt{N}$.
3. Time to solution is calculated by $T_{\text{soln}}=T_{\text{ann}}\times\frac{\log\left(0.01\right)}{\log(1-p_{s})}$, where $T_{\text{ann}}=N_{rt}\times T_{rt}$ is the annealing time, $p_{s}$ is the success probability^12^. Time to the solution of the DOPO-based Ising machine is calculated with *T_ann_* = 11.2 ns, assuming the cavity supported 56 spins, the clock frequency is 5 GHz, and 300 roundtrips are used for computation^8^. As we can see, even though the proposed Ising machine has a lower clock frequency than that of the DOPO-based machine (250 MHz vs. 5 GHz), a shorter time to solution is still obtained.
4. The proposed and the DOPO-based Ising machines solved the max-cut problem of a Möbius ladder graph with 56 vertexes. The memristor-based HNN solved the max-cut instance with 60 vertexes and 50% connectivity, provided in Biq Mac library^6^.
5. Since the spins in superconductor-, LC-oscillator-, SRAM-based Ising machines are locally connected, which leads to the need for a large number of physical spins to implement dense problems. Consequently, the success probability for dense problems decreases rapidly as a function of the size of the problem^12^.

To solve real-world combinatorial optimization problems, the Ising machines should be large-scale, stable, and programable. A large-scale spin network provides enough space for coding complex real-world problems. In the DOPO- and OEPO-based Ising machines, by using the low-loss fiber to store the spins, the number of spins can be as large as 100,000 and is not limited.

Currently, the number of physical spins can be as large as 5,000/60,000 based on superconductor/SRAM. With the development of integration technology, a larger scale of spins can be expected. However, the all-to-all spin interaction is hard to implement in a large network because of the well-known physical constraints on hardware architecture. Spins are deposited in local physical space and interact by short-range specific links (local connectivity). To solve the connectivity problem, several physical spins act as a single logical Ising spin by making the spins on hardware ferromagnetically coupled, which is called “graph embedding”^17^. As a result, the number of logical spins is much less than that of the physical spins. To solve a max-cut problem with *M* vertices, M^2^/d physical spins are usually needed, where *d* is the available degree of a physical spin. For example, although the DW2Q quantum annealer has 2048 physical qubits, no more than 90 logical qubits can be obtained to implement the full connectivity since the embedding introduces considerable overhead. On the other hand, compared to the fully connected Ising model, the graph embedding scheme slows down the computing speed and takes a longer time to reach the ground state^18^. Moreover, when solving the dense graphs, the weaker logical couplings in the graph embedding scheme would hinder the machine’s ability to find the lower Ising energy^19^. Massive spatial free-space multiplexing approaches based on SLM can support a very large number of spins. However, the refresh rate of the SLM is inherently slow^14^.

Currently, limited by fabrication imperfections, the scale of the integration-photonic-based Ising machine is very small. The massive control in Mach-Zehnder interferometers for matrix multiplication is also hard to implement, especially in the large-scale photonic network. For instance, fabrication imperfections in phase setting and beam splitters will lead to errors in the coupling between spins, thus, reducing the efficiency of the algorithm.

The CIMs based on DOPO and OEPO can easily be fully connected using the measurement-feedback scheme with the help of high-speed electric devices, e.g., the FPGA array. One roundtrip time can be considered as one computational step. With a fixed clock frequency, the number of spins is proportional to the cavity delay. On the other hand, the time for one computational step may increase nonlinearly since the matrix computation in the FPGA scales in O(*N*^2^). However, computational time linear to the problem size can still be expected by parallelization technology^9^. Hence, we believe the time for one computational step is linear to the problem size.

Under our current setup, the clock frequency is limited by the bandwidth of the microwave BPF in the OEPO cavity. A higher clock frequency can be expected by using BPFs with a larger bandwidth. Nevertheless, the DOPO can obtain more spins than our proposal if a cavity of the same length is used, as shorter pulse width and higher clock frequency can easily be implemented in the optical domain. Note that the microwave photonic spin can endure greater delay fluctuation than that in DOPO-based CIMs; thus, a longer OEPO cavity can be implemented to support a large scale. According to , in a 5-km fiber loop, a 2.5-femtosecond delay fluctuation or 20-kHz optical frequency variation leads to a *π*-phase change, in other words, a spin flip. Under the same time jitter $\Delta t$, the OEPO-based CIM can support a much longer cavity, which can compensate for the limited clock frequency. For instance, if the clock frequency of the DOPO is 10 times that of the OEPO, the same scale Ising network can be implemented by using an OEPO cavity whose length is 10 times that of the DOPO. For a given number of spins, the lower repetition rate of OEPOs does result in the need for a longer feedback loop and would lead to a longer computation time. Assuming that a certain number of roundtrips is required to solve a combinatorial optimization problem, the computation time is linearly related to the cavity delay. However, since microwave photonic spins are insensitive to uncertain disturbances that would shape the Ising energy landscape and consequently impair the calculation efficiency, the proposed machine needs fewer roundtrips to converge and can eventually reach lower energy.

Compared to the DOPO spin, the microwave photonic spin can tolerate a much larger cavity perturbation due to its longer wavelength. In addition, local microwave oscillation is usually locked to an atomic clock and has no frequency drift. Therefore, the accuracy and stable phase can be obtained in the cavity of the OEPO. The accurate and stable spin phase can also be obtained in the Ising machine based on an LC oscillator since the oscillation frequency is about dozens of kilohertz. However, LC oscillator networks are also limited by physical constraints, similar to the DQ2W. Several physical spins act as a single logical spin to realize the all-to-all networks.

The optoelectronic feedback loop is another scheme to obtain excellent stability since the spin is represented by a baseband pulse with binary amplitudes by utilizing the nonlinearity of the MZM^20^. Currently, the number of spins is very limited, and the clock is very low. In this network, noise is generated digitally, MZM acts as the activation function, and the spin interaction is implemented in the digital domain. From this point of view, the optoelectronic feedback loop is more like a continuous Hopfield neural network, rather than an OEO. This calls into question whether the principle of minimum loss is still applicable to this machine. These similarities and differences between those annealing schemes are an interesting question, and much remains to be studied.

# ****References and notes****

1. Asadi-Zeydabadi, M. Bessel Function and Damped Simple Harmonic Motion. *J. appl. math. phys* **2**, 26–34 (2014).

2. Iannelli, M. *Mathematical theory of age-structured population dynamics*. (Giardini editori e stampatori, 1995).

3. Marki. Triple-balanced mixer. https://www.markimicrowave.com/Assets/datasheets/M2-0020.pdf (2010).

4. Hamerly, R. *et al.* Topological defect formation in 1D and 2D spin chains realized by network of optical parametric oscillators. *Int. J. Mod. Phys. B* **30**, 1630014 (2016).

5. Böhm, F. *et al.* Understanding dynamics of coherent Ising machines through simulation of large-scale 2D Ising models. *Nat. Commun.* **9**, 1–9 (2018).

6. NEOS Server for Optimization. https://neos-server.org/neos/ (2020).

7. Inagaki, T. *et al.* Large-scale Ising spin network based on degenerate optical parametric oscillators. *Nat. Photon.* **10**, 415–419 (2016).

8. McMahon, P. L. *et al.* A fully programmable 100-spin coherent Ising machine with all-to-all connections. *Science* **354**, 614–617 (2016).

9. Honjo, T. *et al.* 100,000-spin coherent Ising machine. *Sci. Adv.* **7**, eabh0952 (2021).

10. Wang, T., Wu, L. & Roychowdhury, J. New Computational Results and Hardware Prototypes for Oscillator-based Ising Machines. in *DAC ’19: The 56th Annual Design Automation Conference 2019* 1–2 (ACM, Las Vegas NV USA, 2019).

11. Russell, J. D-Wave Previews Next-Gen Platform; Debuts Pegasus Topology; Targets 5000 Qubits. https://www.hpcwire.com/2019/02/27/d-wave-previews-next-gen-platform-debuts-pegasus-topology-targets-5000-qubits/ (2019).

12. Hamerly, R. *et al.* Experimental investigation of performance differences between coherent Ising machines and a quantum annealer. *Sci. Adv.* **5**, eaau0823 (2019).

13. Cai, F. *et al.* Power-efficient combinatorial optimization using intrinsic noise in memristor Hopfield neural networks. *Nat. Electron.* **3**, 409–418 (2020).

14. Pierangeli, D., Marcucci, G. & Conti, C. Large-Scale Photonic Ising Machine by Spatial Light Modulation. *Phys. Rev. Lett.* **122**, 213902 (2019).

15. Ramey, C. Silicon Photonics for Artificial Intelligence Acceleration : HotChips 32. in *2020 IEEE Hot Chips 32 Symposium (HCS)* 1–26 (IEEE, Palo Alto, CA, USA, 2020).

16. Takemoto, T., Hayashi, M., Yoshimura, C. & Yamaoka, M. 2.6 A 2 ×30k-Spin Multichip Scalable Annealing Processor Based on a Processing-In-Memory Approach for Solving Large-Scale Combinatorial Optimization Problems. in *2019 IEEE International Solid- State Circuits Conference - (ISSCC)* 52–54 (IEEE, San Francisco, CA, USA, 2019).

17. Choi, V. Minor-embedding in adiabatic quantum computation: II. Minor-universal graph design. *Quantum Inf. Process.* **10**, 343–353 (2011).

18. Miki, T. *et al.* Computational Properties of Ising Spin Model on Spin Connection Parameters. in *2019 IEEE 19th International Conference on Nanotechnology (IEEE-NANO)* 121–124 (IEEE, Macao, 2019).

19. Venturelli, D. *et al.* Quantum Optimization of Fully Connected Spin Glasses. *Phys. Rev. X* **5**, 031040 (2015).

20. Böhm, F., Verschaffelt, G. & Van der Sande, G. A poor man’s coherent Ising machine based on opto-electronic feedback systems for solving optimization problems. *Nat. Commun.* **10**, 3538 (2019).
